# Bifunctional Pd-Al:SrTiO_3_ photocatalyst sheet for m^2^-scale waste PET photoreforming and feasibility study

**DOI:** 10.1039/d6ee01445c

**Published:** 2026-07-08

**Authors:** Ariffin Bin Mohamad Annuar, Yongpeng Liu, Chen Han, Motiar Rahaman, Erwin Reisner

**Affiliations:** a Yusuf Hamied Department of Chemistry, University of Cambridge Lensfield Road Cambridge CB2 1EW UK reisner@ch.cam.ac.uk motiar.rahaman@uliege.be

## Abstract

Photocatalytic reforming of waste plastics offers a route towards a circular plastics economy and sustainable energy generation by producing commodity chemicals and green H_2_ while mitigating plastics accumulation in the environment. As a monomer of polyethylene terephthalate (PET), a dominant waste plastic, ethylene glycol (EG) has gained attention as a promising substrate for reforming processes, but photocatalytic EG oxidation suffers from low product selectivity, slow reaction rates, and poor stability due to the limited availability of efficient cocatalysts. Here, a bifunctional Pd nanoparticle cocatalyst is introduced on an Al-doped SrTiO_3_ (Al:SrTiO_3_) semiconductor to simultaneously promote EG oxidation and H_2_ evolution reactions. Considerations for Pd utilisation at scale in photocatalytic technologies is supported by its projected increase in availability. The Pd-decorated Al:SrTiO_3_ system was coated onto glass substrates to fabricate scalable photocatalytic sheets for a 1 m^2^-scale outdoor demonstration of waste PET bottle reforming. In three days of continuous operation under natural sunlight, 48, 45, 19 and 3 mmol m^−2^ of H_2_, formate, glycolate and glycolaldehyde, respectively, were produced. A comprehensive feasibility study was performed based on this prototype waste PET reforming system to identify key areas to prioritise in future research. Although photocatalytic reforming was unsurprisingly found to be premature for practical application, reactor cost and photocatalyst stability were identified as critical issues to realise a feasible large-scale system in the future. This work presents a robust bifunctional photocatalyst system and provides a systematic feasibility assessment of large-scale PET reforming.

## Abstract 2

A bifunctional Pd catalyst was developed for simultaneous H_2_ evolution and waste polyethylene terephthalate reforming. The practical deployment of the system was demonstrated with an outdoor 1 m^2^ prototype.

## Graphical abstract

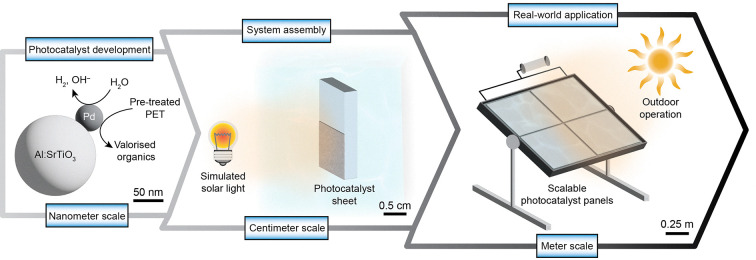

**Broader context**

The photocatalytic reforming of polyethylene terephthalate (PET) waste requires efficient systems for oxidation of its monomer ethylene glycol (EG), which commonly suffers from slow reaction kinetics, poor product selectivity and inadequate long-term stability. Nevertheless, cocatalyst−semiconductor systems capable of efficiently driving both this oxidation half-reaction and the complementary H_2_ evolution half-reaction remain underdeveloped, thereby hindering scale-up and practical implementation. This work introduces a bifunctional metal cocatalyst decorated over a semiconductor material for both photocatalytic PET reforming and H_2_ evolution processes. The rate and selectivity of EG oxidation and H_2_ evolution were enhanced, resulting in charge-balanced product evolution between the half-reactions, thus confirming the completion of the overall photoredox process. Finally, a 1 m^2^ prototype reactor demonstrated scalable waste commercial PET photoreforming under outdoor conditions. Data from the outdoor demonstration were used to perform an in-depth feasibility study using standardised metrics based on techno-economic and life cycle frameworks, and establishes a benchmark for photocatalytic reforming systems.

## Introduction

Plastics have become an intrinsic part of human life, with plastic production projected to increase from around 414 Mt in 2023 to over 1100 Mt in 2050.^1,2^ Considering that the vast majority of plastics are derived from fossil fuels, this rising demand will continue to be a major source of CO_2_ emissions and is projected to account for 20% of global oil use by 2050.^3,4^ Although the need for proper waste plastic management is clear, the strategies employed remain inadequate, with only about 10% of plastic being recycled while the remainder is disposed of *via* landfills, open dumping, incineration or other energy-intensive, emissions-generating approaches such as gasification and pyrolysis.^5–7^ Compounding this issue, rising global H_2_ demand, which is primarily met by carbon-intensive processes such as steam methane reforming or coal gasification,^8,9^ adds further CO_2_ emissions to those already associated with plastic production and treatment. There is thus great interest in alternative technologies that can both upcycle plastics into valuable chemicals and produce sustainable H_2_.

Solar reforming, encompassing photocatalytic, photoelectrochemical and photovoltaic-biased electrochemical reforming, is the solar-driven conversion of waste substrates into value-added chemicals.^10^ In these approaches, the oxidation of organic waste compounds is coupled to a reduction half-reaction, such as the H_2_ evolution reaction (HER), to avoid the sluggish and challenging oxidation of water. This results in a significantly more thermodynamically-favourable reaction for solar reforming (*e.g.*, C_*x*_H_*y*_O_*z*_ + (2*x* − *z*)H_2_O → (2*x* − *z* + ½*y*)H_2_ + *x*CO_2_; Gibbs free energy change, Δ*G*° ≈ 0 kJ mol^−1^) than overall water splitting (H_2_O → H_2_ + ½O_2_; Δ*G*° = +237 kJ mol^−1^).^10,11^ Solar reforming reactions can produce H_2_ together with oxygenates, which are valuable clean energy vectors and precursors in chemical industries.^12–16^ Waste oxidation can also be coupled to CO_2_ reduction to mitigate anthropogenic CO_2_ emissions and potentially achieve a carbon-neutral energy cycle.^17–19^ Of the mentioned solar reforming methods, photocatalytic reforming represents the simplest, integrated method for waste treatment and solar chemical production.

Photocatalytic reforming of plastics, mainly polyethylene terephthalate (PET) with its monomer ethylene glycol (EG) has been widely reported using transition metal oxides such as TiO_2_, metal sulphides, carbon nitrides and many other photocatalytic systems.^20–23^ This shows that PET, constituting 12% of global solid waste,^24^ is a suitable substrate for photocatalytic reforming following pre-treatment to yield EG.^25^ However, the difficulty in EG reforming lies in achieving selective oxidation as formate, glycolate, glycolaldehyde, CO and CO_2_, among others, are potential oxidation products. The majority of EG reforming reports utilise these substrates mainly as electron donors with less emphasis on the characterisation of products from the oxidation half-reaction, leaving potentially valuable oxidation products unaccounted for, as indicated by typically unbalanced charge between oxidation and their reduction half-reaction counterpart.^26^ The incorporation of appropriate cocatalysts is also often necessary for appreciable product formation. A decent number of cocatalysts for the EG oxidation reaction (EGOR) such as Pt, Pd, Au, Cu, Ni, *etc.*, have been reported, particularly for electrochemical processes.^27^ However, their role in photocatalytic systems has not yet been thoroughly investigated, an omission that we address in this study. Meanwhile for the photocatalytic HER, metal cocatalysts such as Pt, Pd, Rh, Ru, Cu, *etc.*, as well as many molecular cocatalysts have been reported.^28^ Pd, capable of promoting both the EGOR and HER, appears to be uniquely suited to couple these half-reactions photocatalytically through a single bifunctional cocatalyst.

The high cost and limited availability of noble-metal cocatalysts have driven interest in finding alternatives.^29^ However, aside from the clear performance advantages of noble-metal cocatalysts, a strong case can be made for specifically developing Pd cocatalysts. Pd demand, driven predominantly by the automotive industry as a catalyst in catalytic converters, is projected to drop drastically (as much as 20% by 2030) due to the increased adoption of electric vehicles.^30^ This will create a surplus of Pd due to the inelastic nature of Pd supply (over 80% of Pd is mined as a by-product of Pt, Ni or Cu and will be produced regardless of market demand).^30,31^ Additionally, as Pd recovery is a relatively mature field, with existing reports on Pd extraction from aqueous waste streams and direct utilisation in catalysis,^32,33^ there are already strategies in development for the circular utilisation of Pd. Hence, using Pd to drive the EGOR and HER justifiably offers a potential pathway for practical photocatalytic reforming.

In transitioning towards industrial applications, the distinction between scalability and feasibility is emphasised. Several noteworthy reports on m^2^-scale photocatalytic systems already exist, which utilise various configurations for scale-up, including photocatalyst (PC) panels, floatable sheets and PC slurries.^34–37^ The use of solar concentration to enhance photocatalytic performance while reducing the use of PC material has also been explored.^38^ Efforts to scale up photocatalytic systems have been accompanied by cost analyses. However, these analyses often rely on unrealistic assumptions, particularly regarding photocatalytic performance and efficiency.^39,40^ Hence, systematic evaluation of the practical feasibility of these systems using standard frameworks such as techno-economic analysis (TEA) and life cycle analysis (LCA) is still required. Photocatalytic systems are not yet commercially feasible in real-world scenarios, but outdoor testing paired with accurate feasibility studies will guide future research directions toward real-world application.

Herein, we introduce Pd as a bifunctional cocatalyst in photocatalytic waste PET reforming to valorised chemicals as well as H_2_ evolution and rationally assess the feasibility of this system on a m^2^-scale guided by TEA and LCA principles. Specifically, the PC system consists of an Al-doped SrTiO_3_ (Al:SrTiO_3_) semiconductor light absorber (selected for its excellent photostability, efficient charge separation and scalable fabrication) loaded with Pd nanoparticles produced by chemical reduction of a Na_2_PdCl_4_ precursor using NaBH_4_. This Pd-loaded Al:SrTiO_3_ powder is then deposited on fluorine-doped tin oxide (FTO) coated glass substrates to form the PC sheets. High resolution scanning transmission electron microscopy with energy-dispersive X-ray spectroscopy, along with *in situ* time-resolved infrared spectroscopy and photoelectrochemical impedence spectroscopy provide strong evidence on the bifunctional role of Pd in enhancing the performance of both the HER and EGOR, as well as improving product selectivity. Building upon promising results from 1 cm^2^ lab-scale experiments, high-throughput spray coating was finally used to fabricate large-scale PC panels on frosted glass for a 1 m^2^ outdoor demonstration of pre-treated waste commercial PET bottle reforming. The data collected from the applied system were used to evaluate its real-world feasibility based on several standardised metrics derived from TEA and LCA. This work demonstrates the potential of Pd as a bifunctional cocatalyst for real-world waste PET reforming and green H_2_ generation, as well as establishes a basis for subsequent efforts to scale up photocatalytic processes.

## Results and discussion

### Synthesis and characterisation of PC powder and sheet

**Fig. 1:**
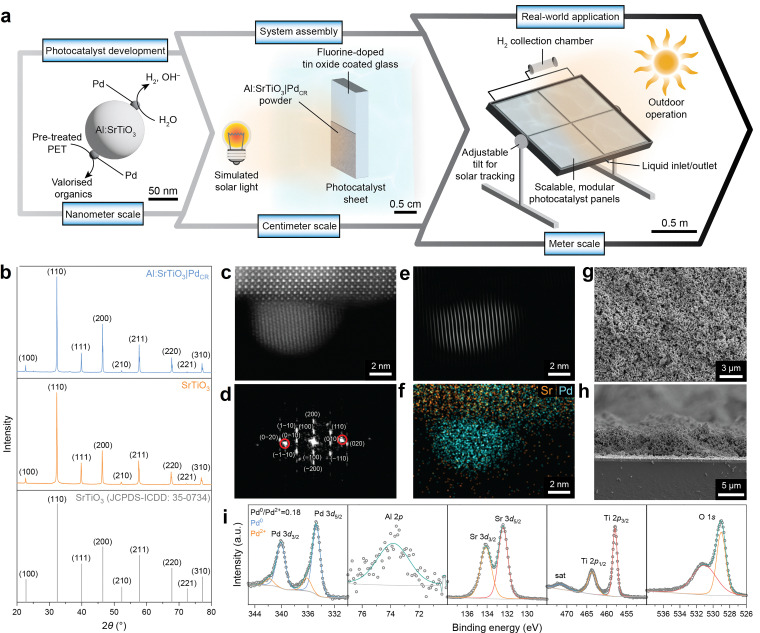
Development and characterisation of the Al:SrTiO_3_|Pd_CR_ photoreforming system. (a) Schematic diagram illustrating the development of the photoreforming system from Al:SrTiO_3_|Pd_CR_ powder into an outdoor waste PET photoreforming system. (b) PXRD patterns for Al:SrTiO_3_|Pd_CR_ PC powder, SrTiO_3_ powder and standard pattern of SrTiO_3_ (JCPDS-ICDD: 35-0734). (c) Atomic-resolution HAADF-STEM image of a Pd nanoparticle on Al:SrTiO_3_|Pd_CR_. (d) Corresponding FFT pattern obtained from the HAADF-STEM image. (e) Inverse FFT of the region of the FFT pattern bounded in red circles. (f) Sr and Pd EDX elemental mapping. (g) Top-view SEM images of Al:SrTiO_3_|Pd_CR_ PC sheet. (h) Cross-section SEM images of Al:SrTiO_3_|Pd_CR_ PC sheet. (i) Curve-fitted XPS spectra of as-prepared Al:SrTiO_3_|Pd_CR_ PC sheets. Sat., satellite peak.

Al:SrTiO_3_ was selected as the light absorber due to its established simple synthesis, excellent quantum yield and high photocatalytic activity, low cost and stability (see Note S1, SI).^41,42^ The Al:SrTiO_3_ powder was synthesised on a gram scale using a previously reported SrCl_2_-flux-mediated Al-doping procedure (see Experimental section).^43^ For the deposition of the Pd cocatalyst from a Na_2_PdCl_4_ precursor, a chemical reduction method was adapted from the literature.^44,45^ Pd was selected due to its potential to act as both H_2_ evolution and EG oxidation catalyst, as indicated by its well-studied application in these reactions.^28,46^ The facile and scalable fabrication of this Al:SrTiO_3_ loaded with chemically-reduced Pd (hereafter referred to as Al:SrTiO_3_|Pd_CR_) allows for the systematic scale up of the PC system from nm-sized powders to cm^2^-scale PC sheets and finally to m^2^-scale PC panels for outdoor application (Fig. 1a).

The powder X-ray diffraction (PXRD) pattern of Al:SrTiO_3_|Pd_CR_ contain the characteristic reflections of SrTiO_3_ (JCPDS-ICDD: 35-0734) as observed previously (Fig. 1b).^47^ A slight peak shift towards higher 2*θ* values also indicated the successful doping of Al (Fig. S1, SI).^48^ This PXRD data, along with the atomic-resolution high-angle annular dark-field scanning transmission electron microscopy (HAADF-STEM) image of Al:SrTiO_3_|Pd_CR_ (Fig. 1c) confirm the highly-crystalline cubic perovskite structure of Al:SrTiO_3_. On the characterisation of the deposited Pd, the atomic-resolution HAADF-STEM image shows that the Pd nanoparticle has well-resolved (111) lattice fringes, which have been reported to be more effective at capturing photogenerated charges.^49^ The corresponding Fast Fourier transform (FFT) pattern from the HAADF-STEM image indicates that the Al:SrTiO_3_ is in polycrystalline form (Fig. 1d). The diffraction spots related to Pd were also reliably assigned by performing inverse FFT on specific regions (Fig. 1d and 1e), and from energy dispersive X-ray (EDX) spectroscopy elemental mapping of the HAADF-STEM image (Fig. 1f), further verifying the crystal structure of the deposited Pd. Additionally, it can be seen from low-magnification transmission electron microscopy (TEM) images and TEM-EDX elemental mapping that the Pd nanoparticles ∼5 nm in diameter were uniformly deposited on the Al:SrTiO_3_ light absorber (Fig. S2 and S3, SI).

For the fabrication of PC sheets, the Al:SrTiO_3_|Pd_CR_ powders were dispersed in isopropanol and deposited using drop casting (PC loading of 2 mg cm^−1^) onto FTO-coated glass as a robust support for forming mechanically stable PC films.^50^ The attachment of the PC powder to the supports was enhanced by annealing the PC sheets at 300 °C in an inert (Ar) atmosphere to prevent further oxidation of the material. The deposited Al:SrTiO_3_|Pd_CR_ formed a uniform layer as observed with scanning electron microscopy (SEM) and SEM-EDX elemental mapping (Fig. 1g and h; Fig. S4, SI). Pre-catalysis X-ray photoelectron spectroscopy (XPS) of the PC sheets showed that Pd species were present in the 0 and 2+ oxidation states (due to surface PdO formation from aerial oxidation) and that each of the other expected elements was present (Fig. 1i).

### Photocatalytic reforming of EG and waste PET

The Al:SrTiO_3_|Pd_CR_ PC sheets (with an active area of 1 cm^2^) were first tested for the photocatalytic reforming of pure EG and waste commercial PET (from waste PET plastic bottles) under lab conditions to better study the PC system in a controlled environment prior to outdoor application (see below). These small-scale photocatalytic experiments were conducted in an air-tight glass photoreactor equipped with a quartz window (solution volume: 12 ml, headspace: ∼12 ml) under simulated solar irradiation (1 Sun, AM1.5G, 100 mW cm^−2^) for 24 h at 25 °C with stirring (Fig. S5, SI).

**Fig. 2:**
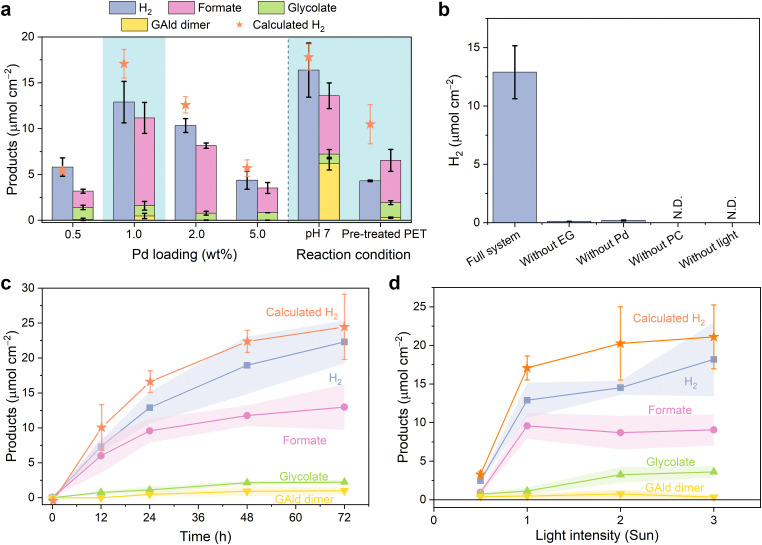
Photoreforming performance of Al:SrTiO_3_|Pd_CR_ PC sheets. (a) Performance of Al:SrTiO_3_|Pd_CR_ PC sheets with different Pd loadings, at different pH and using pre-treated waste commercial PET bottles as the oxidation substrate. Data highlighted in light blue indicate the optimal Pd loading that was subsequently used in experiments with varying reaction condition. (b) Exclusion control experiments showing H_2_ evolution from the full photocatalytic reforming system and with certain components of the system removed. (c) Long-term performance of Al:SrTiO_3_|Pd_CR_ PC sheets. (d) Product evolution at different light intensities. Lines added as a guide to the eye. Calculated H_2_ is the expected amount of H_2_ produced stoichiometrically based on the amount of oxidation products generated. Conditions: photocatalytic experiments were performed under AM1.5G illumination for 24 h (72 h in the case of the long-term experiments in panel c) at 25 °C with stirring. Unless otherwise stated, 0.1 M EG was used as the oxidation substrate in all experiments. N.D., not detected.

The optimal Pd loading on Al:SrTiO_3_ was first determined by varying the deposited Pd from 0.5−5.0 wt%. Inductively coupled plasma optical emission spectroscopy (ICP-OES) showed that the actual Pd loadings agreed well with these calculated values, indicating the successful deposition of Pd by the chemical reduction method (Table S1, SI). For performance evaluation, EG as the model substrate of PET was used as the photocatalytic reforming material. The experiments were performed in 1.0 M aqueous KOH (∼pH 14) to match the conditions of the pre-treated PET substrate that will be used in later experiments. The optimal Pd loading was determined to be 1.0 wt%, which displayed the highest H_2_ evolution of 12.9 ± 2.3 µmol cm^−2^ after 24 h (Fig. 2a) and was thus used in subsequent experiments. Accordingly, PC sheets with this Pd loading also yielded the most EG oxidation products consisting of 9.6 ± 1.7, 1.1 ± 0.5, and 0.5 ± 0.3 µmol cm^−2^ of formate, glycolate and glycolaldehyde (GAld) dimer, respectively (Table S2, SI). With a high selectivity of 86%, the main oxidation product from this PC system is therefore formate, an important energy vector and carbon building block in chemical synthesis.^51^ It was found that lower and higher Pd loadings proved detrimental to photocatalytic performance likely due to lack of active sites for catalysis in the former case and light shielding by dark-coloured Pd in the latter case (Fig. S6, SI). Exclusion control experiments omitting each component of the PC system confirmed that all components are necessary for the photocatalytic activity (Fig. 2b and Table S3, SI). UV−Vis diffuse reflectance spectroscopy measurements and photocatalytic experiments under *λ* > 420 nm irradiation also excluded the possibility of plasmonic effects from the Pd cocatalyst, as the Al:SrTiO_3_|Pd_CR_ showed no response beyond the UV region (Fig. S7, SI).

Other oxidation products, such as CO and CO_2_ from over-oxidation of EG, along with O_2_ from water oxidation were not detectable using gas phase Fourier transform infrared (FTIR) spectroscopy and a fluorescence oxygen sensor probe (Fig. S8 and S9, SI). Quantification of the reduction and oxidation products from the photocatalytic reaction showed a good charge balance between the HER and EGOR half-reactions (Fig. S10 and Table S4, SI). This is reflected in the good agreement between the measured H_2_ and the calculated H_2_ evolution (*i.e.*, expected H_2_ evolution calculated based on oxidation products; Fig. 2a and c). The charge balance between the reduction and oxidation reactions improved with reaction time (deviation between measured and calculated H_2_ of 25% *versus* 10% in the first and last 24 h, respectively; Fig. 2c; Fig. S11 and Tables S5 and S6, SI). This closely-balanced electron–hole consumption between the HER and EGOR processes is important, particularly because most reported solar reforming systems have yet to examine or confirm the charge balance between half-reactions.^52,53^ Detailed explanation on the charge balance calculation and its implications can be found in Note S2 (SI).

The Al:SrTiO_3_|Pd_CR_ PC sheets also possess good stability, as shown in product evolution after 72 h (Fig. 2c and Table S5, SI). It is expected that the lack of CO evolution contributed to the stability of the PC sheets as CO poisoning of the Pd cocatalyst (a known challenge in EG electro-oxidation)^54^ was avoided. Post-catalysis ICP-OES measurements show no significant leaching of Pd after the reactions (Table S1, SI). Scanning transmission electron microscopy (STEM) and SEM images, along with EDX elemental mapping show that there was no noticeable change in both the distribution of Pd nanoparticles on Al:SrTiO_3_ and in the morphology of the PC sheets after catalysis (Fig. S12 and S13). Hence, the loss in performance towards the end of these long-term experiments was possibly caused by poisoning of active sites by build-up of reaction intermediates such as 2-hydroxyacetyl (see below).^54^ The effect of pH on the reaction kinetics was also studied. While pH 14 was initially chosen to match the conditions of alkaline pre-treated PET, the PC sheets were also tested in pH 7 to consider scenarios in which the pre-treated PET solution is neutralised (to remove terephthalate (TPA), a co-product of PET alkaline hydrolysis) prior to photocatalytic reforming. The oxidation product distribution changed from mainly formate (86%) at pH 14 to an even mix of formate and GAld dimer (47% and 46%, respectively) at pH 7 (Fig. 2a and Table S7, SI). This may be due to the difference in reaction pathway of EG oxidation at different pH (see below). Glycolate remained a minor product (<10%) at both pH values.

Towards outdoor validation of the Al:SrTiO_3_|Pd_CR_ PC sheets, experiments were also performed under different simulated solar light intensities, *i.e.*, 0.5−3.0 Sun. The enhancement in photocatalytic performance was less pronounced at higher light intensities (Fig. 2d and Table S8, SI), which can be explained by light-intensity dependent recombination of photogenerated charges.^55^ Using waste commercial PET bottles, pre-treated using alkaline hydrolysis, in place of pure EG monomer, yielded 4.3 ± 0.1, 4.6 ± 1.2, 1.6 ± 0.2 and 0.3 ± 0.1 µmol cm^−2^ of H_2_, formate, glycolate and GAld dimer, respectively (Fig. 2a and Table S9, SI). While the use of EG derived from real-world waste PET as the reforming substrate is encouraging, we observed a 67% drop in performance compared to the experiments with fresh EG. This was consistent with previous reports and is attributed to the competitive adsorption of TPA present in the pre-treated PET solution on active sites which inhibits the adsorption of EG.^51,56^ It is also notable that charge balance was still maintained under the various reaction conditions applied, with the exception of experiments using the pre-treated PET substrate (Fig. S10 and S14; Tables S10–S12; Note S2, SI).

### Pd as a bifunctional cocatalyst for both HER and EGOR

**Fig. 3:**
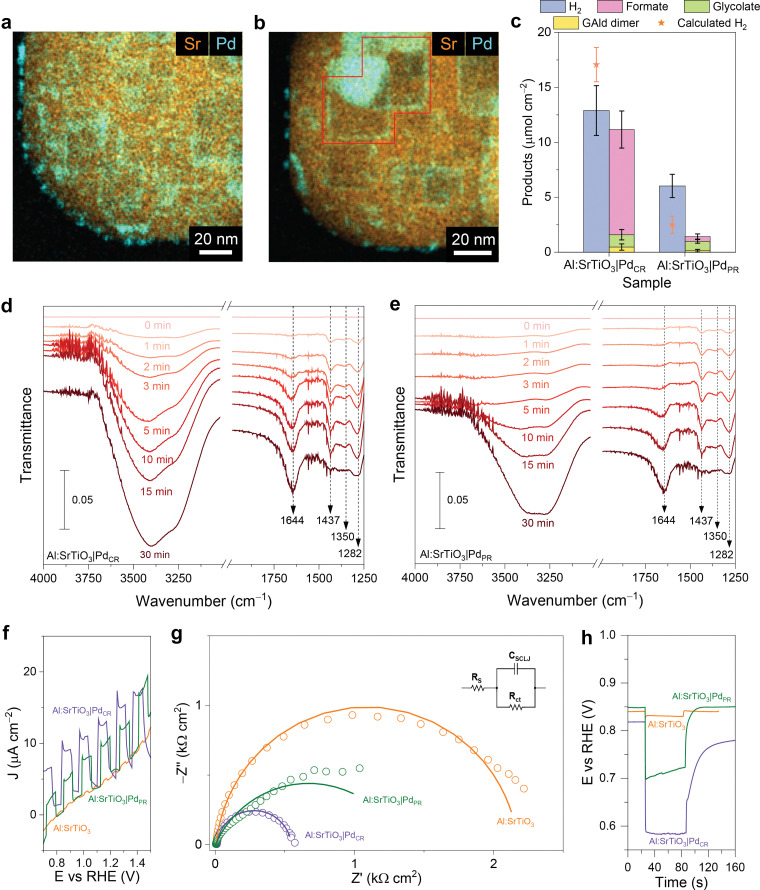
Bifunctional role of Pd cocatalyst. (a) Sr and Pd EDX elemental mapping of Al:SrTiO_3_|Pd_CR_. (b) Sr and Pd EDX elemental mapping of Al:SrTiO_3_|Pd_PR_. Red borders highlight Pd accumulation primarily on the electron-rich (100) facets of Al:SrTiO_3_|Pd_PR_. (c) Product evolution from Al:SrTiO_3_|Pd_CR_ and Al:SrTiO_3_|Pd_PR_ PC sheets. (d) *In situ* ATR-IR spectra of EG photoreforming by Al:SrTiO_3_|Pd_CR_ at pH 14. (e) *In situ* ATR-IR spectra of EG photoreforming by Al:SrTiO_3_|Pd_PR_ at pH 14. All spectra have been background-subtracted at *t* = 0 min in the dark, after which the sample was illuminated. (f) Chopped light linear sweep voltammograms of Al:SrTiO_3_|Pd_CR_, Al:SrTiO_3_|Pd_PR_ and bare Al:SrTiO_3_. (g) Nyquist plots of Al:SrTiO_3_|Pd_CR_, Al:SrTiO_3_|Pd_PR_ and bare Al:SrTiO_3_ recorded at 1 V *versus* reversible hydrogen electrode (RHE) with frequency ranges from 2.2 kHz to 0.5 Hz and a 15 mV sinusoidal AC perturbation (open circles) as well as the corresponding fitting curves (solid lines). Inset: proposed Randles equivalent circuit, *C*_SCLJ_ was replaced by a constant phase element (CPE) during fitting to account for the non-ideal capacitive behaviours of the photoelectrodes. (h) Chopped light open circuit potentials of Al:SrTiO_3_|Pd_CR_, Al:SrTiO_3_|Pd_PR_ and bare Al:SrTiO_3_. Conditions: PEC measurements were performed in 30 ml stirred, N_2_-saturated 1.0 M KOH (pH 14) electrolyte containing 0.1 M EG under AM1.5G illumination. Photocatalytic experiments were performed in 1.0 M KOH (pH 14) containing 0.1 M EG under AM1.5G illumination for 24 h at 25 °C with stirring.

To elucidate the role of Pd as a bifunctional cocatalyst for both the HER and EGOR, the performance of the Al:SrTiO_3_|Pd_CR_ system was compared to the same PC system with Pd loaded using photoreduction (hereafter referred to as Al:SrTiO_3_|Pd_PR_). Due to the anisotropic charge separation in Al:SrTiO_3_ upon illumination,^42^ photoreduction of Pd allows for the preferential accumulation of Pd on the electron-rich facets of the Al:SrTiO_3_, thus promoting the HER. Atomic-resolution HAADF-STEM images and EDX elemental mapping confirmed that Pd was evenly distributed on both the (100) and (110) facets of Al:SrTiO_3_|Pd_CR_ but mainly on the (100) electron-rich facets of Al:SrTiO_3_|Pd_PR_ (Fig. 3a and b; Fig. S15 and S16, SI).^42^ The EDX elemental mapping along with XPS verify the presence of all expected elements for both PC samples (Fig. 1i; Fig. S17−S19, SI). Quantitative XPS fitting shows that the Sr 3d_5/2_:3d_3/2_ ratios for Al:SrTiO_3_|Pd_CR_ and Al:SrTiO_3_|Pd_PR_ were 1.2 and 1.3, respectively. On the other hand, their Ti 2p_3/2_:2p_1/2_ ratios were both 1.6 (Fig. 1i and Fig. S19, SI). These similar ratios indicated that both Al:SrTiO_3_|Pd_CR_ and Al:SrTiO_3_|Pd_PR_ possess a similar amount of oxygen vacancies and that the use of NaBH_4_ for the chemical reduction of Pd onto Al:SrTiO_3_|Pd_CR_ did not introduce unintended structural modifications which could have affected photocatalytic performance.

Al:SrTiO_3_|Pd_PR_ was assembled as PC sheets following the same procedure as Al:SrTiO_3_|Pd_CR_ above and the performances of the two PC sheets were directly compared. The Al:SrTiO_3_|Pd_CR_ sheets displayed a photocatalytic performance more than double that of the Al:SrTiO_3_|Pd_PR_ sheets (H_2_ evolution of 12.9 ± 2.3 and 6.0 ± 1.1 µmol cm^−2^ in 24 h, respectively; Fig. 3c and Table S13, SI). The external quantum efficiency (EQE) at 350 nm was 4.7% for Al:SrTiO_3_|Pd_CR_ and only 1.8% for Al:SrTiO_3_|Pd_PR_. It is noted that Al:SrTiO_3_ PC systems can achieve higher EQE but require more complex synthesis procedures involving multiple cocatalysts.^42^ Both PC sheets had a similar Pd loading (0.86 *versus* 0.91 wt% for Al:SrTiO_3_|Pd_PR_ and Al:SrTiO_3_|Pd_CR_, respectively; Table S1, SI), but the photoreduced Pd may have a lower active surface area compared to the chemically-reduced Pd due to uneven accumulation of Pd on only specific Al:SrTiO_3_ facets. This could partially contribute to their differing performances in these and following experiments comparing the PC systems with different Pd deposition methods. Nevertheless, with an EG oxidation product generation of 0.4 ± 0.2, 0.9 ± 0.2 and 0.1 ± 0.1 µmol cm^−2^ of formate, glycolate and GAld dimer, respectively, Al:SrTiO_3_|Pd_PR_ also lost product selectivity (86% selectivity to formate in the case of Al:SrTiO_3_|Pd_CR_; Fig. 3c and Table S13, SI). Thus, the lack of Pd on the oxidation facets of Al:SrTiO_3_|Pd_PR_ proved detrimental to performance and Pd therefore promotes both the HER and EGOR in the Al:SrTiO_3_|Pd_CR_ system.

To further understand the bifunctional role of Pd, the EG oxidation mechanism of Al:SrTiO_3_|Pd_CR_ and Al:SrTiO_3_|Pd_PR_ was studied using *in situ* attenuated total reflection infrared (ATR-IR) spectroscopy (Fig. 3d, e and Fig. S20). The ATR-IR spectra of both PC systems showed similar features, with bands at 1437 and 1282 cm^−1^ attributed to adsorbed EG and the band around 3250−3500 cm^−1^ attributed to adsorbed OH species from water, KOH or EG (peak assignment can be found in Table S14, SI). More interestingly, the band at 1644 cm^−1^, assigned to 2-hydroxyacetyl, indicates that the EGOR for both PC proceeds *via* a well-known pathway from GAld (which can self-dimerise under the reaction conditions)^57^ to glycolate and finally to formate through the initial formation of the 2-hydroxyacetyl intermediate (Fig. S21, SI).^58,59^ Finally, the band at 1350 cm^−1^ can be assigned to produced formate and glycolate species. This band remained small throughout the measurement, indicating the relatively fast desorption of the formed products. There was also the absence of bands associated with adsorbed CO species (∼2050, ∼1850 and ∼1830 cm^−1^ for linear, bridge and multi-bound CO, respectively; Fig. S20).^59,60^ In combination, these data suggest that for the Al:SrTiO_3_|Pd_CR_ system, the Pd cocatalyst enables the oxidation of EG primarily to formate and glycolate, which desorbs without further oxidation to CO_2_ or CO. The difference in mechanism at different pH is also addressed in Note S3 (SI). Additionally, 2-hydroxyacetyl intermediate peaks form later with Al:SrTiO_3_|Pd_PR_ than Al:SrTiO_3_|Pd_CR_ (∼5 *versus* ∼2 min, respectively), suggesting slower reaction kinetics, in line with the measured photocatalytic performance in Fig. 3c. This further supports that the Pd cocatalyst was beneficial not only for H_2_ evolution, but also for EG oxidation.

The difference in photocatalytic performance between Al:SrTiO_3_|Pd_CR_ and Al:SrTiO_3_|Pd_PR_ PC sheets was then probed by photoelectrochemical (PEC) measurements. The PEC properties of the sheets were assessed by fabricating photoelectrodes on FTO-coated substrates (see Experimental section for details). Linear sweep voltammetry (Fig. 3f) shows a negligible photocurrent density for pristine Al:SrTiO_3_ (*ca.* 0.1 µA cm^−2^) in presence of EG, indicating poor charge extraction and a lack of catalytic activity. Upon introduction of Pd, the anodic photocurrent densities, which are characteristic of the n-type semiconducting behaviour of Al:SrTiO_3_,^61^ increased by more than an order of magnitude, reaching 9 µA cm^−2^ for Al:SrTiO_3_|Pd_CR_ and 5 µA cm^−2^ for Al:SrTiO_3_|Pd_PR_. The photocurrent behaviour was further examined by chronoamperometry (Fig. S22), which confirms stable photocurrent under the applied conditions and demonstrates that the films are functional photoelectrodes. It is noted that due to the fundamentally different operating principles of photocatalytic and PEC systems, the nanoparticulate Al:SrTiO_3_|Pd_CR_ architecture is not optimised for efficient charge extraction, meaning that the PEC measurements presented here display low current density and are not directly comparable with other PEC systems in the literature.^62^

Photoelectrochemical impedance spectroscopy (PEIS) shows that deposition of Pd by chemical reduction (for Al:SrTiO_3_|Pd_CR_) and photoreduction (for Al:SrTiO_3_|Pd_PR_) substantially lowers the charge-transfer resistance (*R*_ct_) from 2.2 to 0.5 and 1.3 kΩ cm^−2^, respectively (Fig. 3g and Table S15, SI). These PEIS results support the conclusion that the Pd cocatalyst facilitates interfacial charge transfer^63^ with chemically-reduced Pd being more effective than photoreduced Pd, as reflected by the lower *R*_ct_ of the former compared to the latter. Open circuit potential measurements (Fig. 3h) reveal photovoltages of approximately 10 mV for pristine Al:SrTiO_3_, 230 mV for Al:SrTiO_3_|Pd_CR_ and 140 mV for Al:SrTiO_3_|Pd_PR_. The significantly enhanced photovoltage is indicative of suppressed charge recombination and improved charge separation.^64^ Together, these PEC characterisations demonstrate that while both Pd-loaded PC systems outperformed the pristine Al:SrTiO_3_ light absorber for EG oxidation, Al:SrTiO_3_|Pd_CR_ (with Pd evenly distributed on oxidation and reduction facets of Al:SrTiO_3_) had superior performance to Al:SrTiO_3_|Pd_PR_ (with Pd mostly deposited on reduction facets of Al:SrTiO_3_).

### Outdoor photocatalytic waste PET reforming at m^2^-scale

**Fig. 4:**
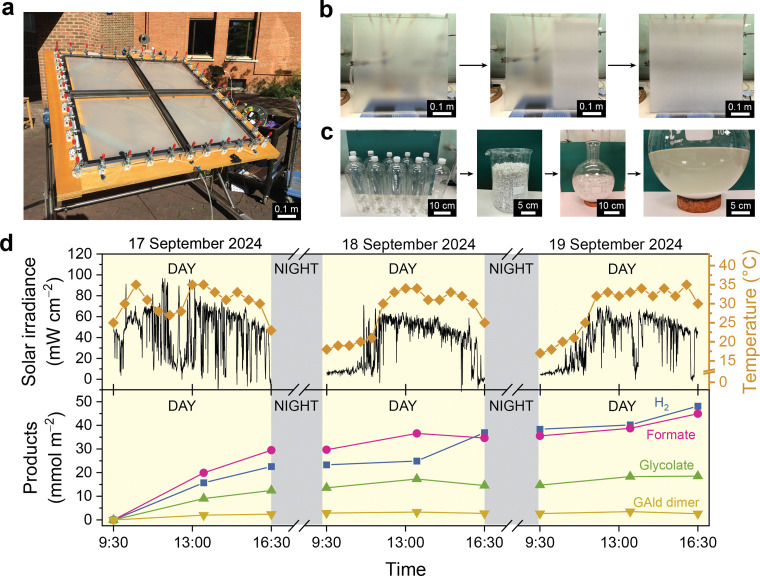
1 m^2^ demonstration of commercial waste PET bottle photoreforming under outdoor conditions. (a) Photograph of the large-scale 1.4 m × 1.4 m photoreactor during operation.^36^ (b) Fabrication of Al:SrTiO_3_|Pd_CR_ PC panels by spray coating. (c) Stepwise pre-treatment process of waste commercial PET bottles. (d) Weather conditions, sunlight intensity and atmospheric temperature (top panel) and product formation with time (bottom panel) over the course of the 1 m^2^ outdoor demonstration.

The simple and scalable fabrication of the Al:SrTiO_3_|Pd_CR_ system, as well as its robust performance in lab-scale tests facilitate its application in the large-scale reforming of waste commercial PET bottles. To that end, a previously reported^36^ custom air-tight 1.4 m × 1.4 m area panel photoreactor was used (Fig. 4a). The photoreactor consisted of a main reforming chamber (total volume of ∼25 litres) with an ultraviolet-transparent acrylic window supported by a mobile base which can be tilted to better track the sun. Gas samples were collected in a separate chamber. The photoreactor housed four 0.25 m^2^ PC panels prepared by high-throughput spray coating of the PC onto frosted glass panels (Fig. 4b). The sheets fabricated in this way had similar light absorption and photocatalytic performance as those fabricated by drop casting, as confirmed by small-scale photocatalytic experiments comparing the deposition methods which yielded similar H_2_ evolution (Fig. S23). For the reforming feedstock, pre-treated waste commercial PET bottles, obtained from a local market, were used (Fig. 4c). The plastic bottles were first shredded to fine flakes. To simplify solution handling, the PET flakes were digested in 4.0 M KOH solution at 80 °C under stirring for 3 days before being diluted to 1.0 M KOH prior to the outdoor demonstration. The 1 m^2^ outdoor PET reforming demonstration was performed continuously for three days from 17 to 19 September 2024 in front of the Yusuf Hamied Department of Chemistry, with weather conditions (incident solar irradiance and ambient temperature) recorded throughout the demonstration (Fig. 4d, top panel). Further details on experimental setup and conditions are outlined in Note S4 (SI).

Due to the intermittent nature of natural sunlight and conducting the experiment in early autumn in Cambridge, the average light intensity over the three-day demonstration was only ∼0.4 Sun. Nevertheless, the photocatalytic reforming products still evolved relatively steadily with time, reaching a total of 48, 45, 19 and 3 mmol m^−2^ of H_2_, formate, glycolate and GAld dimer, respectively, at the end of the experiment (Fig. 4d, bottom panel; Table S16, SI). In the first few hours of the experiment, evolved H_2_ gas was trapped in the solution, causing H_2_ production to appear lower than expected. Larger H_2_ bubbles then form and could be seen flowing upwards throughout the demonstration (Video S1). While the Al:SrTiO_3_|Pd_CR_ PC panels could maintain their performance for extended durations, as indicated by only a small drop in product evolution rate over the three-day demonstration (partly attributable to variations in weather conditions), the demonstration was concluded after three days due to unfavourable weather conditions. Nevertheless, based on this outdoor demonstration, the performance of the present Al:SrTiO_3_|Pd_CR_ PC panels was still comparable to state-of-the-art photocatalytic reforming systems (Table S17, SI). It should also be noted that most reported systems were only tested under lab conditions and at mg or cm^2^ scale. The merit of the Al:SrTiO_3_|Pd_CR_ system becomes even clearer considering the production of value-added organic chemicals, the use of waste commercial PET and the operation of the present system on a m^2^-scale under natural sunlight for a relatively long duration. The result from our large-scale demonstration (covered in more detail in Note S5, SI) showed that the Al:SrTiO_3_|Pd_CR_ PC system is suitable for outdoor application.

### Feasibility study of 1 m^2^ PET reforming system

While large-scale photocatalytic reforming and water splitting have been reported,^34,35^ in-depth feasibility studies on such systems are rare (the state of feasibility studies in the field is described in Note S6, SI), thus limiting the insights that can be gained from these large-scale demonstrations. Hence, measured data from the 1 m^2^ demonstration using Al:SrTiO_3_|Pd_CR_ was used to perform a rational, well-founded feasibility study informed by a TEA and LCA framework (Tables S18−S21, SI) to comprehensively evaluate the system as a benchmark for future works. The findings of the feasibility study were summarised in three standard metrics, namely solar-to-value (STV) creation rate, energy return on investment (EROI) and carbon footprint (see Note S7 in SI and Experimental section for detailed methodology and equations). The STV creation rate is a relatively recent metric referring to the economic value creation from the system considering products, reactants, as well as capital, operating and consumables costs.^10^

**Fig. 5:**
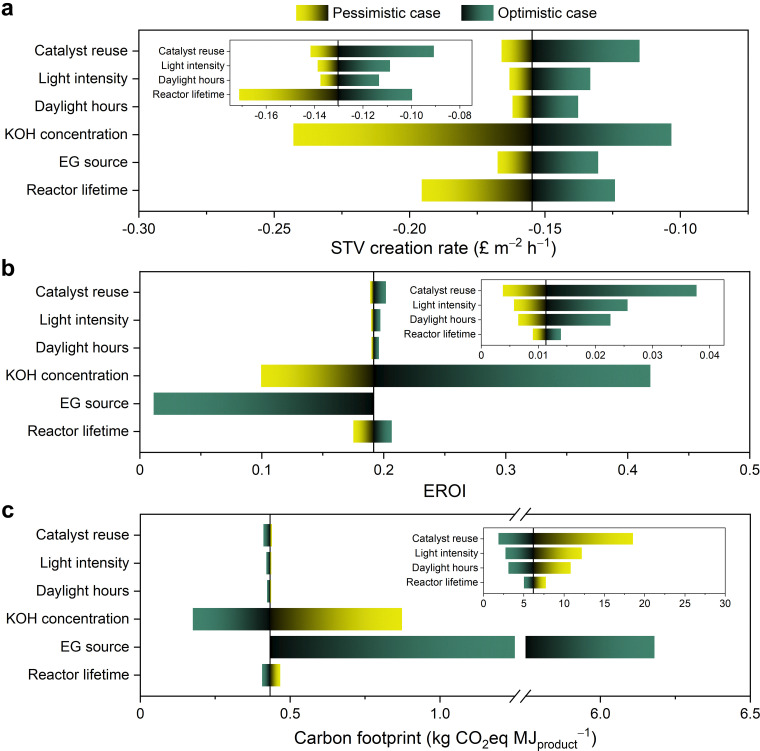
Feasibility of waste PET photoreforming. (a) Sensitivity analysis of STV creation rate of the overall waste PET photoreforming process. (b) Sensitivity analysis of EROI of the overall waste PET photoreforming process. (c) Sensitivity analysis of carbon footprint of the overall waste PET photoreforming process. Insets: Corresponding STV creation rate, EROI and carbon footprint considering only photocatalytic aspects (*i.e.*, without considering waste PET pre-treatment). The values were calculated by varying individual parameters between “optimistic” and “pessimistic” estimates and based on results obtained from the 1 m^2^ outdoor demonstration using Al:SrTiO_3_|Pd_CR_.

The overall Al:SrTiO_3_|Pd_CR_ PET reforming system had a STV creation rate, EROI and carbon footprint of −0.15 £ m^−2^ h^−1^, 0.19 and 0.43 kg CO_2_eq MJ_products_^−1^, respectively (Fig. 5a–c). The STV creation rate and EROI were both lower than their respective breakeven points of zero and one (exceeding these breakeven points would indicate a net economic and energy gain from the reforming system). The carbon footprint of the system was also higher than that of conventional waste disposal strategies.^65^ These results were expected, as photocatalytic reforming systems currently operate at efficiencies well below the threshold needed for commercial use. The intention of this feasibility study is therefore to identify key aspects to prioritise in future development of photocatalytic reforming systems.

To do this, a sensitivity analysis was performed by recalculating the feasibility metrics after varying selected reaction parameters, namely catalyst reuse (*i.e.*, stability), light intensity, daylight hours, KOH concentration, EG source and reactor lifetime, from the “base” case (*i.e.*, the actual experimental conditions) to an “optimistic” or “pessimistic” case (Fig. 5a–c and Tables S22–S25, SI). The “optimistic” and “pessimistic” cases for these parameters were set based on realistic, achievable scenarios. While the costs associated with product separation from the PET reforming system was not considered in the present study, considerations for this challenge are discussed in Note S8 (SI). A base-level analysis of the results shows that factors related to PET pre-treatment (KOH concentration and EG source) dominated the overall PET reforming process. This was expected because the alkaline hydrolysis of PET is a more developed technology than photocatalytic reforming. The use of KOH for PET pre-treatment also had a relatively large impact on the EROI and carbon footprint of the overall PET reforming system (Tables S24 and S25, SI). Hence, other methods of PET pre-treatment should also be explored in the future, such as acidic PET hydrolysis, which offers high PET hydrolysis rates,^66,67^ as well as enzymatic PET hydrolysis, which can be performed under comparatively mild conditions while maintaining high PET hydrolysis performance.^68^ Interestingly, when sourcing EG from EG-containing effluent in the “optimistic” case, there was an obvious economic benefit as seen in the increase in STV creation rate. However, the EROI and carbon footprint became more unfavourable (these metrics respectively decreased and increased) due to the loss of solid waste upcycling and environmental remediation aspects. This suggests that economic and environmental considerations do not necessarily align, reflecting the reality that sustainable practices often impact profitability.

To examine the relative effect of only the photocatalytic process on the feasibility of the overall PET reforming system, a set of feasibility metrics were calculated excluding the PET pre-treatment process (Fig. 5a–c inset and Tables S26–S28, SI). Reactor lifetime has the highest effect on STV creation rate due to the high cost of building the photoreactor. This agreed with the findings of previous TEA reporting that capital expenditure accounts for the majority of the total cost of a modelled large-scale photocatalytic system.^40^ When excluding the capital costs, the STV creation rate approached the breakeven point, reaching as high as −0.03 £ m^−2^ h^−1^ in “optimistic” cases (Fig. S24 and Table S29, SI). This emphasises the role of economic incentives from governing bodies in potentially implementing emerging solar technologies, such as solar reforming, which have a high upfront investment cost. Catalyst reuse (*i.e.*, stability) was also an important economic factor, with the STV creation rate being significantly higher in the “optimistic” case where the PC was reused more times, requiring less replacement. The same parameter was also the most impactful for EROI and carbon footprint. This was attributed to the relatively energy-intensive and environmentally impactful synthesis of chemicals for PC preparation. Light intensity and daylight hours also had a significant influence on all feasibility metrics, emphasising the importance of selecting an ideal location for solar chemical production.

Finally, the Al:SrTiO_3_|Pd_CR_ PET reforming process was compared to a hypothetical overall water splitting process using the same PC and with the same photocatalytic performance (Fig. S25−S27 and Tables S30−S32, SI). The STV creation rate of the hypothetical water splitting process is still similar to that of the PET reforming process (−0.15 £ m^−2^ h^−1^) due to the substantial cost contribution of PET pre-treatment. However, considering only the photocatalytic component, the STV creation rate became higher for the PET reforming process (−0.13 £ m^−2^ h^−1^; Table S26, SI) than the water splitting process. This was because valorised organics were produced instead of relatively less valuable O_2_ in the respective processes. It is also apparent that solar H_2_ production without complementary organics production from waste upcycling had a considerably reduced EROI and increased carbon footprint (∼2 orders of magnitude of difference in EROI and carbon footprint), highlighting the potential advantages of solar reforming over solar water splitting, particularly in terms of energy efficiency and environmental remediation. It is additionally expected that the STV creation rate, EROI and carbon footprint of the photocatalytic reforming process can be further improved by replacing the HER with a CO_2_ reduction reaction to potentially produce more complex chemicals, although this would require further development of the PC system (see Note S9, SI).

## Conclusions

This study introduces a bifunctional Pd catalyst in a photocatalytic system for scalable sunlight-driven waste PET reforming. The Pd, loaded onto an Al:SrTiO_3_ light absorber by simple chemical reduction, allows the Al:SrTiO_3_|Pd_CR_ PC sheets to perform H_2_ evolution along with EG oxidation to formate with high selectivity with glycolate and GAld dimer as relatively minor side products. Well-balanced charge consumption between the HER and EGOR half-reactions confirmed that all significant EG oxidation products were accounted for, thus completing the overall solar-driven reaction. The bifunctional role of Pd was further validated by photocatalytic experiments exploiting the anisotropic charge separation property of Al:SrTiO_3_, as well as by atomic-resolution HAADF-STEM, EDX spectroscopy, *in situ* ATR-IR spectroscopy and PEC techniques including PEIS. A 1 m^2^ outdoor demonstration of photocatalytic waste PET reforming was performed, owing to the facile, scalable fabrication of Al:SrTiO_3_|Pd_CR_ PC panels using high-throughput spray coating. The photocatalytic reforming feedstock was sourced from waste commercial PET bottles pre-treated using alkaline hydrolysis, thus combining sustainable organics production and solid waste treatment. The outdoor demonstration showed continuous product formation over three days, with the potential for extended operation under favourable weather conditions. Considering the photocatalytic performance, outdoor conditions, scale and value-added oxidation products, the present Al:SrTiO_3_|Pd_CR_ system compares favourably to existing EG/PET photocatalytic reforming systems.

Using data obtained from the outdoor demonstration, the feasibility of the complete PET pre-treatment and photocatalytic reforming process was assessed based on multidimensional feasibility metrics, namely STV creation rate, EROI and carbon footprint. The EG source and KOH concentration had the largest effect on the feasibility metrics. Focusing only on the photocatalytic component of the overall process, catalyst reuse and photoreactor lifetime become key parameters affecting the metrics, while light intensity and daylight hours remain important. Hence, advances in catalyst design and reactor engineering will be critical for the development of viable photocatalytic systems. Additionally, the feasibility study showed that photocatalytic reforming could be more promising than overall water splitting, particularly in terms of energy efficiency and environmental remediation. While the feasibility metrics indicate that photocatalytic reforming is not yet ready for commercial application, this feasibility study establishes a benchmark for photocatalytic reforming systems and provides a valuable guideline for future research directions.

The application of a bifunctional catalyst to promote the HER and EGOR in tandem can potentially be a solution to producing sustainable H_2_ and upcycling waste plastics into commodity chemicals. Subsequent work in this area would prioritise optimising the waste pre-treatment procedure, as well as catalyst design for improving stability, because these will be essential factors in developing realisable photocatalytic waste reforming processes, as indicated by the feasibility study.

## Experimental section

### Materials

SrTiO_3_ (99.9%, Bioserv), SrCl_2_·6H_2_O (99.9%, Thermo Scientific), Al_2_O_3_ nanopowder (<50 nm, Sigma-Aldrich), Na_2_PdCl_4_ (98%, Sigma-Aldrich), polyvinylpyrrolidone (average mol wt 40 000, Sigma-Aldrich), NaBH_4_ (≥98.0%, Sigma-Aldrich), 2-propanol (≥99.5%, Sigma-Aldrich), ethylene glycol (≥99%, Sigma-Aldrich), KOH (≥85%, Fisher Chemical), Milli-Q water (18.2 MΩ·cm, Millipore).

### Synthesis of Al:SrTiO_3_

Al:SrTiO_3_ was prepared by a previously reported flux method.^43^ 1.84, 0.020, and 26.7 g of SrTiO_3_, Al_2_O_3_ nanopowder, and SrCl_2_·6H_2_O (molar ratio of 1:0.02:10) were mixed using an agate mortar. The mixture was then heated in an alumina crucible at 1423 K for 10 h and subsequently cooled to room temperature, after which the product was stirred in 500 ml of Milli-Q water. The resulting powder was collected by filtration to remove any impurities associated with the SrCl_2_. This rinsing process was repeated three times. The resulting Al:SrTiO_3_ powder was dried at 313 K overnight.

### Synthesis of Pd-loaded Al:SrTiO_3_

For Al:SrTiO_3_|Pd_CR_, Pd was deposited onto Al:SrTiO_3_ based on a chemical reduction method adapted from the literature.^44^ 100 mg of Al:SrTiO_3_ was dispersed in 10 ml of H_2_O using ultrasonication for 10 min. 94 µl of 0.1 M Na_2_PdCl_4_ and 10 µl of 0.25 mM polyvinylpyrrolidone were added and the mixture (with a final Na_2_PdCl_4_ concentration of 0.94 mM) was sonicated for 10 min and stirred for a further 10 min. 1 ml of 0.13 M NaBH_4_ was added dropwise to the mixture. After a final 20 min of stirring, the prepared Al:SrTiO_3_|Pd_CR_ powder was collected by centrifugation, washed with H_2_O several times followed by ethanol, and dried overnight at room temperature in a N_2_ atmosphere.

For Al:SrTiO_3_|Pd_PR_, Pd was deposited onto Al:SrTiO_3_ by photoreduction. 100 mg of Al:SrTiO_3_ was dispersed in 10 ml of H_2_O using ultrasonication for 10 min. 94 µl of 0.1 M Na_2_PdCl_4_ and 10 µl of 0.25 mM polyvinylpyrrolidone were added and the mixture (with a final Na_2_PdCl_4_ concentration of 0.94 mM) was sonicated for 10 min and stirred for a further 10 min. The mixture was then irradiated in a solar light simulator calibrated to match air mass 1.5 global (AM1.5G) irradiation for 3 h at room temperature. The prepared Al:SrTiO_3_|Pd_PR_ powder was collected and dried using the same method as Al:SrTiO_3_|Pd_CR_.

### Fabrication of small-scale PC sheets

The PC sheets were fabricated by depositing the PC onto FTO-coated glass. The glass sheets were successively washed in water, ethanol and acetone before being dried and placed in an ultraviolet ozone cleaner for 20 min to remove possible organic material from the surface of the sheets. 2.0 mg of PC powder (Al:SrTiO_3_|Pd_CR_ or Al:SrTiO_3_|Pd_PR_) was added to 200 µl of isopropanol and ultrasonicated for 5 min until the catalyst was well-dispersed. This catalyst mixture was drop casted onto the FTO-coated glass in 4 equal layers (50 µl per layer), with the sheets being allowed to dry between each successive layer. After drying, the PC sheets were finally annealed at 300 °C for 1 h in an Ar atmosphere.

### Fabrication of large-scale PC panels

For the fabrication of the 0.25 m^2^ PC panels for the large-scale outdoor demonstration, Al:SrTiO_3_|Pd_CR_ was first dispersed in isopropanol (10 mg ml^−1^) by ultrasonication. The PC dispersion was then spray coated onto 50 cm × 50 cm frosted glass sheets under ambient conditions and without heating using a Paasche H-Series Single Action Suction Feed Airbrush.

### Materials characterisation

A TESCAN MIRA3 FEG-SEM instrument with an Oxford Instruments Aztec Energy X-maxN 80 system was used to collect the SEM images and EDX mapping. A 300 kV Thermo Fisher Scientific Spectra with 4 EDS Super-X detectors was used to collect atomic-resolution HAADF-STEM and accompanying EDX maps. Inductively-coupled plasma optical emission spectrometry (ICP-OES) was carried out on a Thermo Scientific iCAP 700 spectrometer and elemental CHN analysis was obtained using a PerkinElmer 240 Elemental Analyser or an Exeter Analytical CE-440 Elemental Analyser by the University of Cambridge (Department of Chemistry) Microanalysis Service. Powder X-ray diffraction (PXRD) analysis was conducted using a Panalytical X'Pert Pro powder X-ray diffractometer by the University of Cambridge X-ray laboratory. XPS analysis was performed using a Thermo Scientific Escalab 250Xi fitted with a monochromated Al kα X-ray source (1486.7 eV). All data were recorded with an X-ray beam size of 650 µm, a pass energy of 20 eV at a step size of 0.1 eV. Electronic charge neutralisation was achieved using an ion source with ion gun current and voltage of 100 µA and 40 V, respectively. All sample data were recorded at a pressure below 10^−8^ Torr and a room temperature of 294 K.

### *In situ* ATR-IR measurements

*In situ* ATR-IR experiments were conducted using a customised *in situ* photochemical cell. 1 mg of the PC (Al:SrTiO_3_|Pd_CR_ or Al:SrTiO_3_|Pd_PR_) was dispersed in a mixture of 1 ml of isopropanol and 50 µl of 5 wt% Nafion under ultrasonication for 30 min to produce an ink. The prepared ink was then painted onto a Au layer, which was sputter-coated onto a Si prism. A few drops of the reaction solution consisting of 0.1 M EG in 1.0 M KOH or H_2_O was added to the PC layer. The sample was then irradiated with a 5 W 365 nm light source. All spectroscopic measurements were collected at a resolution of 4 cm^−1^ with 128 co-added scans using a PerkinElmer Spectrum 3 FTIR spectrometer equipped with a liquid nitrogen-cooled MCT detector and a VeeMax III accessory.

### PEC and electrochemical measurements

All PEC measurements were conducted under simulated AM1.5G irradiation (1 Sun, 100 mW cm^−2^) using a Newport Oriel 67005 solar light simulator in a 3-electrode configuration with an Al:SrTiO_3_ working electrode, a 1 cm × 1 cm Pt mesh counter electrode, and a Ag/AgCl (satd. NaCl, BASi) reference electrode. The N_2_-saturated KOH (1.0 M, pH 14) electrolyte contains EG (0.1 M). The working electrode on FTO-coated glass was fabricated in the same way as the PC sheet using drop casting. Impedance response was recorded at 1 V *versus* RHE using an IviumStat with frequency ranges from 2.2 kHz to 0.5 Hz and a 15 mV sinusoidal amplitude. Impedance data was fitted with equivalent circuits using modeling software ZView2 (Scribner Associates).

Cyclic voltammograms were measured using an IviumStat potentiostat in a 3-electrode configuration with a Pd-deposited carbon foil working electrode, a Pt mesh counter electrode, and a Ag/AgCl (satd. NaCl, BASi) reference electrode. The N_2_-saturated KOH (1.0 M, pH 14) electrolyte contains EG (0.1 M). The working electrode was fabricated by drop casting Pd nanoparticles onto a carbon foil with Nafion as a binder.

All potentials recorded with the Ag/AgCl (saturated NaCl) reference electrodes were converted to the RHE scale using eqn (1), where *E*_RHE_ and *E*_Ag/AgCl_ are the potentials with respect to RHE and Ag/AgCl (satd. NaCl, BASi) reference electrode, respectively, and pH is the pH of the reaction medium at 298 K.

1 *E*_RHE_(*V*) = *E*_Ag/AgCl_(*V*) + 0.197(*V*) + 0.059(*V*) × pH

### Pre-treatment of real-world waste PET bottles

For lab-scale experiments, the real-world waste PET plastic water bottle was pre-treated using an alkaline hydrolysis method.^68^ In brief, the PET bottle was first cut into small pieces and dipped in liquid nitrogen to embrittle the PET shards. Thereafter, the pieces were shredded using a grinder. The ground PET bottle was then added to 1.0 M aqueous KOH at a concentration of 50 mg ml^−1^ and heated to 80 °C for 3 days under stirring to ensure sufficient depolymerisation of the PET. The solution was then kept unperturbed at room temperature to cool and finally filtered through a polytetrafluoroethylene syringe filter (0.2 µm) to remove unreacted PET. The solution had an EG concentration of 0.14 M, as measured by high performance liquid chromatography (HPLC).

The waste PET bottles for the large-scale outdoor demonstration were pre-treated in a similar method. 10 two-litre-volume waste PET plastic water bottles were cut into small pieces, yielding ∼200 g of PET shards. After freezing in liquid nitrogen and shredding, the PET was depolymerised in 6 litres of 4.0 M KOH at 80 °C for 3 days under stirring. The pre-treated solution was then passed through a fine-mesh sieve to remove larger unreacted PET and finally diluted 4× to obtain a 1.0 M KOH solution with 0.025 M of EG.

### Photocatalytic experiments in lab (1 cm^2^) scale

Photocatalytic measurements were conducted in a side-irradiation-type glass photoreactor equipped with a quartz window. For photocatalytic reforming experiments, 12 ml of the chosen feedstock (0.1 M EG or pre-treated PET) was added to the photoreactor. The prepared PC sheets were then placed into the photoreactor and held in place by a stainless-steel T-rod support. The liquid and photoreactor headspace were purged with N_2_ (containing 2% CH_4_ for gas chromatographic analysis) for 30 min. All purging needle holes were then sealed with Loctite superglue Universal adhesive. The sheets were then irradiated by a solar light simulator (Newport Oriel 67005) calibrated to match AM1.5G irradiance. Unless otherwise mentioned, the experiments were conducted at 25 °C with stirring.

### Large-scale (1 m^2^) waste PET bottle reforming

For the large-scale experiments, four Al:SrTiO_3_|Pd_CR_ panels were placed into a custom-built 1.4 m × 1.4 m area panel photoreactor previously reported (Fig. 4a; a detailed description of the photoreactor and 1 m^2^ demonstration is provided in Note S4, SI).^36^ The photoreactor was clamped shut and filled with 20 litres of pre-treated PET using a submersible water pump. The liquid and photoreactor headspace were purged with N_2_ (containing 2% CH_4_ for gas chromatographic analysis) for 30 min using tubing installed at the bottom and top of the photoreactor.

The photoreactor was then placed under natural sunlight for 3 days (7 h of sunlight irradiation per day) in front of the Yusuf Hamied Department of Chemistry. The panels were left immersed in the pre-treated solution in the sealed photoreactor throughout the 3 day experiment. The photoreactor was tilted and rotated to track the sun while incident light intensity was continuously measured using a Newport 843-R-USB Handheld Power Meter equipped with a 919P-020-12 Thermopile sensor. Gas products were collected in a separate gas collection chamber connected to the headspace of the photoreactor while liquid products were collected by drawing liquid samples from the photoreactor.

### Product analysis

Gas chromatography (GC) was used to measure H_2_ evolution. 50 µl of gas samples were periodically taken from the headspace of the photoreactors and analysed using a Shimadzu GC-2010 Plus gas chromatograph with a barrier discharge ionisation (BID) detector. 2% CH_4_ was used as an internal standard to monitor any gas leakage during experiment. The presence or absence of CO and CO_2_ formation was observed with FTIR using a Thermo Scientific Nicolet iS50 spectrometer. O_2_ evolution was monitored using a NeoFox-GT fluorometer and Fospor-R fluorescence oxygen sensor probe (Ocean Optics). EG, formate, glycolate and GAld dimer concentrations were determined by HPLC on a Waters Breeze system equipped with a RIS-2414 refractive index detector and a Rezex ROA-Organic Acid H^+^ 300 × 7.80 mm HPLC column using 2.5 mM H_2_SO_4_ as the eluent with a flow rate of 0.5 ml min^−1^ at 75 °C. The response factors for the substrates were determined by calibration of aqueous solutions with known amounts of the respective substrates.

### Calculation of EQE

The external quantum efficiency was calculated from photocatalytic H_2_ evolution experiments using eqn (2):2
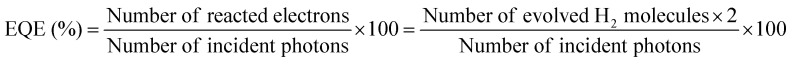


### Feasibility study on large-scale waste PET reforming

The feasibility study was conducted based on data obtained from the large-scale demonstration of PET reforming using the Al:SrTiO_3_|Pd_CR_ PC panels. The metrics selected for the feasibility study were STV creation rate, EROI and carbon footprint. Details on cost, embodied energy and carbon footprint calculations are listed in Tables S18−S21 (SI).

The STV creation rate was calculated using eqn (3),^10^ where *C*_*i*_ is the cost of chemical product *i*, *n*_*i*_ is the amount of chemical product *i*, *C*_op_ is the hourly operating cost, *C*_con_ is the hourly consumables cost, *C*_inv_ is the capital investment cost, *A* is the photoactive area (1 m^2^) and t is the operating time (21 h).3
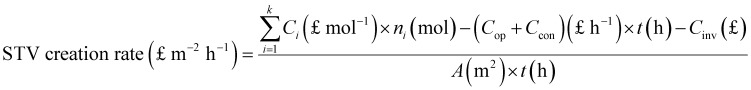


The EROI was calculated using eqn (4),^39^ where *E*_*i*_ is the embodied energy of chemical product *i*, *n*_*i*_ is the amount of chemical product *i*, *E*_op_ is the hourly operating energy, *E*_con_ is the embodied energy of consumables, *E*_inv_ is the embodied energy of capital investment and *t* is the operating time (21 h).4
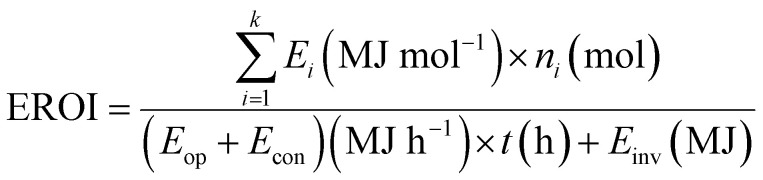


The carbon footprint was calculated using eqn (5),^39^ where *F*_op_ is the carbon footprint of hourly operation, *F*_con_ is the carbon footprint of consumables per hour, *F*_inv_ is the carbon footprint of capital investment, *t* is the operating time (21 h), *E*_*i*_ is the embodied energy of chemical product *i* and *n*_*i*_ is the amount of chemical product *i*.5
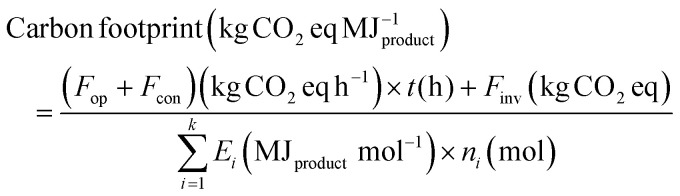


### Statistics

Average values are based on measurements performed in triplicates (*n* = 3), except for the large-scale outdoor experiments, where the values are based on single measurements. The errors correspond to the standard deviation of data points from individual samples.

## Author contributions

A.B.M.A., M.R. and E.R. designed the project. A.B.M.A. performed the photocatalytic reforming experiments and designed the large-scale photoreactor. Y.L. characterised the PC sheet samples and performed photoelectrochemical measurements. C.H. performed ATR-IR spectroscopy measurements. A.B.M.A. performed the feasibility study. A.B.M.A., M.R. and E.R. co-wrote the manuscript with inputs and discussions from all co-authors. M.R. and E.R. supervised the work.

## Conflicts of interest

A patent application covering this work has been filed by Cambridge Enterprise that names A.B.M.A., M.R. and E.R. as co-inventors (GB 2614082.2).

## Supplementary Material

EE-019-D6EE01445C-s001

EE-019-D6EE01445C-s002

## Data Availability

The data underlying this study's findings are accessible through the University of Cambridge data repository: https://doi.org/10.17863/CAM.131028. Supplementary information is available. See DOI: https://doi.org/10.1039/d6ee01445c.
