# Supplementary material for: Bifunctional Pd-Al:SrTiO3 photocatalyst sheet for m2-scale waste PET photoreforming and feasibility study
Source: Energy Environ Sci. 2026 Jul 8;19(15):4983–98. doi: 10.1039/d6ee01445c (PMC13343467; doi:10.1039/d6ee01445c)
Supplement: EE-019-D6EE01445C-s001 [file EE-019-D6EE01445C-s001.pdf]

Supplementary Information (SI) for Energy & Environmental Science.
This journal is © The Royal Society of Chemistry 2026

Electronic Supplementary Information (ESI)

Bifunctional Pd–Al:$SrTiO_3$ Photocatalyst Sheet for m^2-Scale Waste PET Photoreforming and Feasibility Study

Ariffin Bin Mohamad Annuar,[1] Yongpeng Liu,[1] Chen Han,[1] Motiar Rahaman,[1,2,*] and Erwin Reisner[1,*]

[1] *Yusuf Hamied Department of Chemistry, University of Cambridge, Lensfield Road, Cambridge CB2 1EW, UK*

[2] *Current Address: Department of Chemical Engineering, University of Liège, Sart-Tilman, 4000 Liège, Belgium*

* Corresponding authors

*E-mail: reisner@ch.cam.ac.uk (Professor Erwin Reisner)

motiar.rahaman@uliege.be (Dr Motiar Rahaman)

Note S1. Rationale for utilising Al:$SrTiO_3$ for large-scale experiments.

Although Al:$SrTiO_3$ is limited to ultraviolet-light absorption, its excellent photostability, efficient charge separation and scalable fabrication still make it highly attractive for large-scale outdoor photocatalytic applications. Here, the advantages of the Al:$SrTiO_3$ light absorber are discussed.

Firstly, Al:$SrTiO_3$ possesses exceptional chemical and photochemical stability, making it highly attractive for large-scale outdoor application, even more so for photocatalytic reforming under strong alkaline conditions such as in the present work. Many commonly used visible-light-responsive photocatalyst (PC), such as carbon nitrides and metal sulphides, undergo photocorrosion under prolonged irradiation, leading to poor stability.[1,2] The highly alkaline conditions (pH 14) used for PET reforming also complicates the use of other visible-light-absorbing PC such as $BiVO_4$, WO_3, etc., due to dissolution of the PC under these harsh conditions.[3,4] On the other hand, the robust chemical structure of Al:$SrTiO_3$ provides it with high stability, with reports of Al:$SrTiO_3$-based photocatalytic systems maintaining their photocatalytic performance for up to several months.[5,6] This robustness is particularly important for scaled systems intended for continuous outdoor operation, where long-term durability is essential for practical deployment.

In addition to its stability, Al:$SrTiO_3$ exhibits enhanced charge carrier transport and suppressed charge recombination due to Al doping.[7] These substitutional Al dopants inhibit the formation of Ti^{3+} defects which can act as charge recombination sites and enable more efficient utilisation of absorbed photons.[8,9] This has enabled Al:$SrTiO_3$-based PC systems to achieve internal quantum efficiencies approaching 100%.[10] Hence, although Al:$SrTiO_3$ absorbs only the ultraviolet (UV) region of the solar spectrum, it still outperforms many visible-light-absorbing PC systems.[11–13]

Al:$SrTiO_3$ is also scalable due to its relatively simple solid-state flux synthesis procedure, which has allowed this PC to be reliably synthesised on kg-scales.[6,11] Thus, towards the development of a practical photocatalytic reforming system, Al:$SrTiO_3$ is highly applicable for the fabrication of large-scale PC sheets requiring larger amounts of PC material compared to lab-scale system.

While broad-spectrum light utilisation will be important for future outdoor deployment of photocatalytic systems, Al:$SrTiO_3$ is still a good choice for the present work considering the limitations of current visible-light-absorbing materials.

Note S2. Charge balance between the HER and EGOR for Al:$SrTiO_3$|Pd_{CR} and Al:$SrTiO_3$|Pd_{PR} PC sheets.

As organic substrate oxidation can yield multiple liquid and even gaseous products, the charge balance between the reduction and oxidation half-reactions should be calculated to determine the extent to which the oxidation products have been accounted for. For EG oxidation in the present Al:$SrTiO_3$|Pd_{CR} system, the most likely oxidation products based on the known EG oxidation pathway, along with the number of electrons required for the formation of each product is listed in Table S33.

After eliminating the possibility of CO and CO_2 evolution from EG oxidation as well as O_2 evolution from water oxidation by Fourier transform infrared (FTIR) spectroscopy and a fluorescence oxygen sensor probe (Figs. S8 and S9), the only other identified EG oxidation products were formate, glycolate and GAld dimer. From this and the quantified H_2 evolution, the total electron and holes consumed from the HER and EGOR can be calculated (Fig S10 and Table S4). While the charge consumed in these half-reactions roughly match each other, the holes consumed tends to exceed the electrons consumed, indicating that less reduction products (only H_2 in this case) is produced than expected (as can be seen in the comparison between the measured and calculated H_2 in Figure 2a). This can be attributed to electron consumption by the partial reduction of Pd^{2+} to Pd^0 as seen in post-catalysis XPS of the Al:$SrTiO_3$|Pd_{CR} PC sheets possessing a greater Pd^0/Pd^{2+} ratio than the pre-catalysis samples (Figs. 1i and S28). Slight leakage of H_2 over the course of the 24 h experiments can also increase the discrepancy in charge consumption.

In the long-term experiments, the difference between the measured and calculated H_2 decreased with time, becoming insignificant by the end of the 72-h experiment (Fig. 2c). The improvement in charge balance (Fig. S11 and Table S6) was likely due to gradual, more complete reduction of Pd^{2+} to Pd^0 over time, thus removing a potential source of electron consumption. This is why electrochemical processes using Pd cocatalysts typically include an activation step in which residual Pd^{2+} is electro-reduced to Pd^0,[14] a process that understandably requires a longer time in the present purely photocatalytic system.

Unlike other experiments with the Al:$SrTiO_3$|Pd_{CR} PC sheets, the HER and EGOR half-reactions showed a relatively poor charge balance when using pre-treated PET as the substrate, with the apparent number of electrons consumed being outmatched by the holes consumed leading to a lower measured H_2 evolution than expected (Figs. 2a and S10; Table S12). The

relatively low apparent H_2 evolution could be due to H_2 consumption in side-reactions involving PET depolymerisation intermediates, such as ring hydrogenation of terephthalates, which have been reported to occur with Pd catalysts.[15]

On the charge balance of the Al:$SrTiO_3$|Pd_{PR} PC sheets, the calculated H_2 was significantly less than that of the measured H_2 (Fig. 3c). While CO, CO_2 and O_2 measurements apparently showed that none of these oxidation products had evolved (Figs. S29 and S30), it is also possible that CO_2 in particular could be trapped in the reaction solution as the photocatalytic experiments were performed in 1.0 M KOH. Post-catalysis XPS revealed that there was some oxidation of Pd^0 species to Pd^{2+} (Fig. S31), which could also account for the poor charge balance of the Al:$SrTiO_3$|Pd_{PR} sheets (Figs. 3c and S32; Table S34).

Note S3. In situ ATR-IR spectroscopic measurements on Al:$SrTiO_3$|Pd_{CR} during the photocatalytic reforming of EG at pH 14 and 7.

As mentioned, under alkaline conditions EG oxidation on Al:$SrTiO_3$|Pd_{CR} occurs via the 2-hydroxyacetyl intermediate to first form GAld, followed by glycolate and finally formate. Comparing the ATR-IR spectra of EG reforming at pH 14 and 7, the major difference is the less intense peak at 3250–3500 cm^{-1} and the disappearance of the peak at 1644 cm^{-1} (Figs. 3d and S33). The less intense peak at 3250–3500 cm^{-1} is expected due to less adsorbed OH species under neutral conditions. This also relates to the difference in product distribution observed under different pH. In alkaline conditions, higher concentration of adsorbed OH facilitates the deprotonation of EG, thus lowering the barrier of oxidation. So, under neutral conditions, GAld as a less-oxidised product forms to a greater extent.[16,17] Finally, the lack of the 1644 cm^{-1} peak could be caused by quick transformation of the 2-hydroxyacetyl intermediate to GAld.[18]

Note S4. Experimental setup and conditions of the large-scale outdoor demonstration of photocatalytic waste commercial PET bottle reforming.

The 1 m^2 photocatalytic PET reforming demonstration was carried out in a purpose-built panel photoreactor with a 1.4 m × 1.4 m area.[19] The photoreactor consisted of a main reaction chamber fabricated from acrylic with an ultraviolet-transparent window. Inside the chamber, an acrylic crossbeam served both as structural reinforcement and as a support for four Al:$SrTiO_3$|Pd_{CR} PC panels, each with an area of 0.25 m^2, resulting in a total irradiation area of

1 m^2 (Figs. 4a, 4b and S34). An external aluminium frame provided further rigidity. The chamber was mounted on a wooden base and secured with 40 toggle clamps, while a compressed neoprene gasket along the perimeter ensured a gas-tight seal during operation. The reactor was designed with the ability to tilt and rotate for solar tracking and was fitted with liquid and gas ports for purging, solution filling, and product collection. For gaseous products, the chamber was connected via valve-fitted rubber tubing to a detachable collection reservoir.

The feedstock used for the large-scale demonstration consisted of waste commercial PET bottles pre-treated using alkaline hydrolysis. As shown in Figure 4c, 10 large PET bottles were first physically broken down to small PET flakes by cutting and grinding. This was followed by hydrolysis in KOH. To simplify solution handling, the PET flakes were treated in 4.0 M KOH (rather than 1.0 M KOH as used in small-scale PET pre-treatment) at 80 °C for 3 days with stirring. 6 litres of feedstock solution was prepared in a single batch, filtered to remove unreacted PET and then diluted to 24 litres (to dilute KOH to 1.0 M, the same concentration used in the lab-scale PET reforming experiments) for use in the demonstration. This pre-treated PET solution, containing 0.025 M of EG, was pumped into the sealed photoreactor using a circulation pump.

The 1 m^2 demonstration was performed outside the Chemistry of Health building beside the Yusuf Hamied Department of Chemistry continuously for three days from 17−19 September 2024. The position of the sun was tracked throughout the experiment to maximise solar insolation. The incident light intensity and ambient temperature throughout the demonstration were recorded (Fig. 4d, top panel). Liquid samples were drawn from the photoreactor periodically to measure EG oxidation products. On the other hand, evolved H_2 was sampled directly from the gas reservoir.

Note S5. Results of the large-scale outdoor demonstration in the broader context of photocatalytic reforming research.

The large-scale system produced 48, 45, 19 and 3 mmol m^{-2} of H_2, formate, glycolate and GAld dimer, respectively, after three days of operation. A comparison between this and previous systems can be drawn. However, comparing the performance of photocatalytic reforming systems can be challenging as experimental conditions such as light source, reaction scale, duration of experiment, reaction solution, etc., vary between reports. Additionally, the performance of PC systems is evaluated by different metrics, such as product evolution on a

mass or areal basis. Direct conversion between these metrics is also difficult because PC systems are optimised based on a chosen metric. Hence, the literature comparison in Table S17 lists reported PC systems for EG and/or PET reforming as well as relevant experimental conditions and the metric used to evaluate performance.

It can be seen that besides very few exceptions, photocatalytic reforming systems are tested only on small scales, i.e., in the order of mg/μg of PC or cm^2 of active area. Extrapolating these systems to g/kg or m^2 scale will likely overestimate their performance. Performing experiments in ideal lab conditions with powerful light sources and model EG substrate is also favourable to photocatalytic performance and stability. Given these factors, Table S17 includes the performance of the present $Al:SrTiO_3|Pd_{CR}$ system under various conditions (including a suspended powder system; Fig. S35 and Table S35) to enable a more straightforward comparison with reported systems.

The H_2 evolution of the $Al:SrTiO_3|Pd_{CR}$ PC sheets is comparable with high-performing EG/PET reforming systems. On the oxidation side, value-added organics are produced at a high rate and selectivity compared to previous reports. While large-scale PET reforming has been demonstrated before,[20] the quantification of oxidation products is still lacking. The production of organics is an important advantage of the $Al:SrTiO_3|Pd_{CR}$ system as these products are more valuable than H_2 alone. Therefore, the present $Al:SrTiO_3|Pd_{CR}$ system generally compares favourably to existing PC systems. It is noted, however, that photocatalytic reforming systems are still outperformed by benchmark photocatalytic water splitting systems, which in turn have their own scale-up challenges.

Note S6. The current state of feasibility studies on photocatalytic systems.

The overwhelming majority of photocatalytic reforming (and water splitting) systems are tested in lab conditions using photoreactor designs that are difficult to scale up. This is understandable as most research is focused on PC optimisation, rather than developing the system surrounding the PC. The relatively low technological maturity makes conducting feasibility studies challenging, as many subjective assumptions are necessary to fill the gaps in data.[21] Nevertheless, there have been attempts to assess the feasibility of several photocatalytic reforming systems. These studies show that H_2 or valuable organics production by photocatalytic reforming can be economically competitive with conventional production

methods.[22,23] While the prospects of photocatalytic reforming are promising, these feasibility studies are prone to being overly-optimistic.

As large-scale implementation of photocatalytic reforming are rare, all reported feasibility studies have been conducted by scaling up lab-scale systems to industrial scale or by modelling systems.[22–25] This may lead to extremely large errors in the results of the feasibility studies if they are not conducted on a well-grounded premise. Yet, the assumptions made in these studies—primarily on PC efficiency and stability—substantially exceed the achievements of actual systems, and are often several orders of magnitude above even state-of-the-art devices.[26,27] While there is value in these studies in providing a preliminary basis for evaluating PC systems in the real world, the large potential for error may diminish the perceived importance of certain research aspects and bias the trajectory of future research directions.

Note S7. The feasibility study on the Al:$SrTiO_3$|Pd_{CR} system.

As mentioned, the reliability of a feasibility study is dependent on the data upon which it is based. Therefore, this work utilises actual data obtained from the outdoor demonstration of the Al:$SrTiO_3$|Pd_{CR} PC system to produce a more accurate feasibility study on a photocatalytic reforming system. Several established metrics were used to evaluate the system on a standardised basis. These metrics consisted of solar-to-value (STV) creation rate, energy return on investment (EROI) and carbon footprint (see Experimental section for equations).

The scope of this feasibility study was limited to the actual operation and performance of the 1 m^2 photoreactor. This means that the metrics were calculated based on the actual amounts of products generated and the associated capital and operational costs, embodied energy and carbon footprint from the 1 m^2 demonstration (including PET pre-treatment). As such, the only assumption was on photoreactor lifetime (conservatively assumed to be 500 days). To study the relative contributions of several selected reaction parameters, a sensitivity analysis was performed. The parameters of the 1 m^2 demonstration was set as the "base" case. An "optimistic" and "pessimistic" case was then made for each parameter to individually assess the effect each parameter had on each feasibility metric (Table S22). A set of the same feasibility metrics were also calculated for just the photocatalytic reforming process (i.e., excluding PET pre-treatment) so that parameters relevant to photocatalysis can be assessed more clearly. Finally, the Al:$SrTiO_3$|Pd_{CR} system was compared to a similarly performing

hypothetical water splitting system to examine to advantages of waste reforming over pure water splitting.

It is not expected that the present system will be feasible for real-world implementation as the efficiencies of PC systems in general are too low for meaningful application. However, the relative contributions of the main factors affecting the feasibility of photocatalytic reforming can be made clear, allowing the present work to serve as a reliable guide for subsequent research efforts.

Note S8. Considerations for product separation from the PET reforming system.

The major products from the overall PET reforming system are ethylene glycol (EG) and terephthalate (TPA) from the alkaline hydrolysis of PET, as well as H_2 and formate from the subsequent photocatalytic reforming process. H_2 as the only gaseous product can be collected from the headspace of the photoreactor. In the current design of the large-scale panel photoreactor, H_2 is collected in a detachable gas reservoir separate from the main photoreactor cell. As for TPA, neutralisation of the alkaline PET hydrolysis solution will lead to the precipitation of TPA, which can then be recovered by simple filtration.[28] For EG, its separation from water is already performed efficiently on an industrial scale by vacuum distillation[29] enabled by the large difference in boiling points between EG and water (197 and 100 °C, respectively).

On the other hand, the separation of formate from the photocatalytic reforming solution can be more challenging due to the formation of a high boiling azeotrope between formate and water. In this regard, liquid-liquid extraction has been reported to be a cost-effective and relatively less energy-demanding process, particularly compared to conventional distillation.[30,31] Ion exchange can also be used to recover formate from the reaction solution. This method has been shown to be particularly effective for dilute formate streams, as is likely the case in photocatalytic reforming systems.[32,33] Hence, although product separation was not considered in detail in the present work, established methods for addressing this challenge already exist.

Note S9. CO_2 reduction experiments using the Al:$SrTiO_3$|Pd_{CR} PC system.

As Pd is a known CO_2 reduction cocatalyst, the possibility of utilising the Al:$SrTiO_3$|Pd_{CR} system for this reaction was also investigated. However, from the photocatalytic experiments, no gaseous CO_2 reduction products formed as H_2 was the only gas product detected (Fig. S36a). As formate was produced from the PC system and is also a common CO_2 reduction product from Pd catalysts, a $^{13}CO_2$ isotopic-labelling experiment was performed to determine if a portion of the formate produced was sourced from CO_2. Proton nuclear magnetic resonance (^{1}H-NMR) spectroscopy of the reaction solution showed no formation of ^{13}C-formate (Fig. S36b). Hence, it was concluded that CO_2 reduction is not possible using the Al:$SrTiO_3$|Pd_{CR} system in its current configuration as the Al:$SrTiO_3$ generated reductive driving force is not sufficient for the overpotentials required for CO_2 reduction reaction.

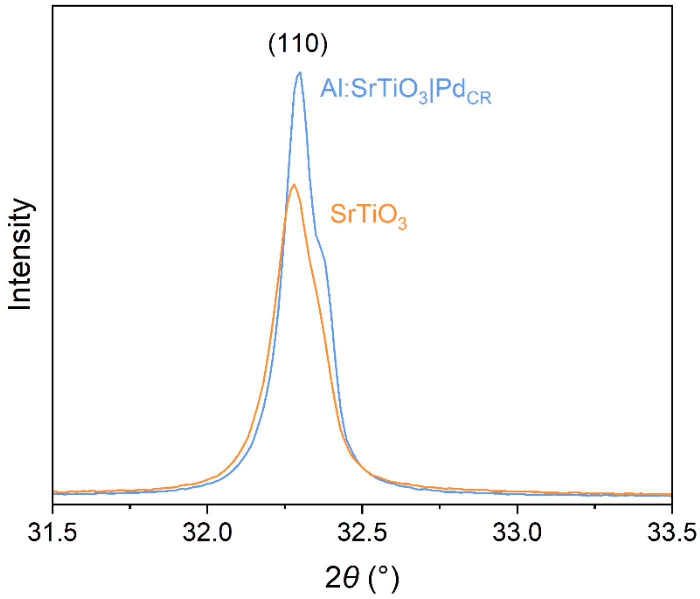

Fig. S1. Enlarged view of PXRD patterns for Al:$SrTiO_3$|Pd_{CR} and $SrTiO_3$. The patterns show a slight doping-induced peak shift.

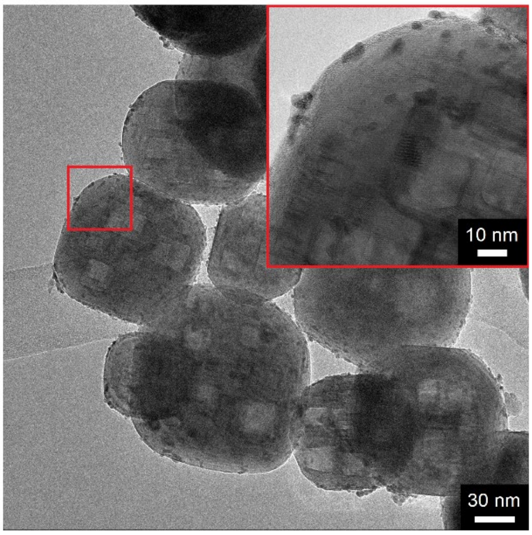

Fig. S2. Low-magnification TEM image of Al:$SrTiO_3$|Pd_{CR} powder. Inset: magnified view of the region bounded by the red square.

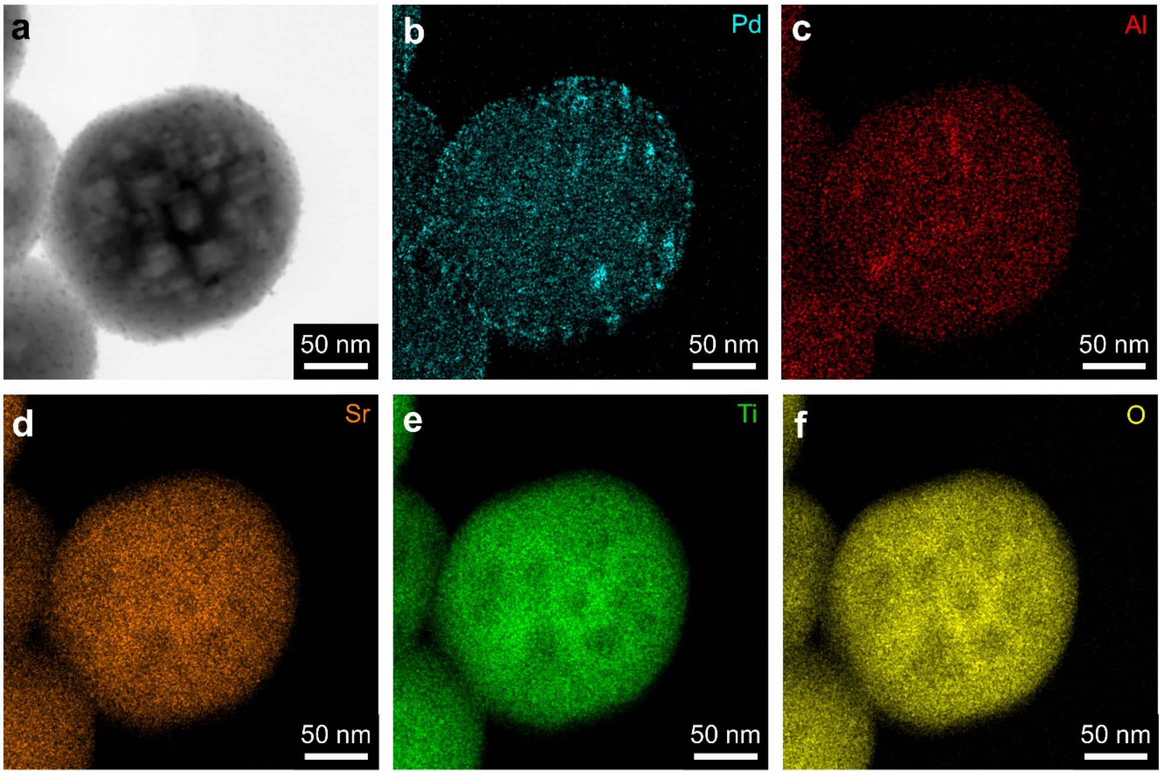

Fig. S3. BF-STEM image and EDX elemental mapping of as-prepared Al:$SrTiO_3$|Pd_{CR}. a, Bright-field STEM image of Al:$SrTiO_3$|Pd_{CR} powder. **b-f,** Corresponding Pd (**b**), Al (**c**), Sr (**d**), Ti (**e**) and O (**f**) EDX elemental mapping.

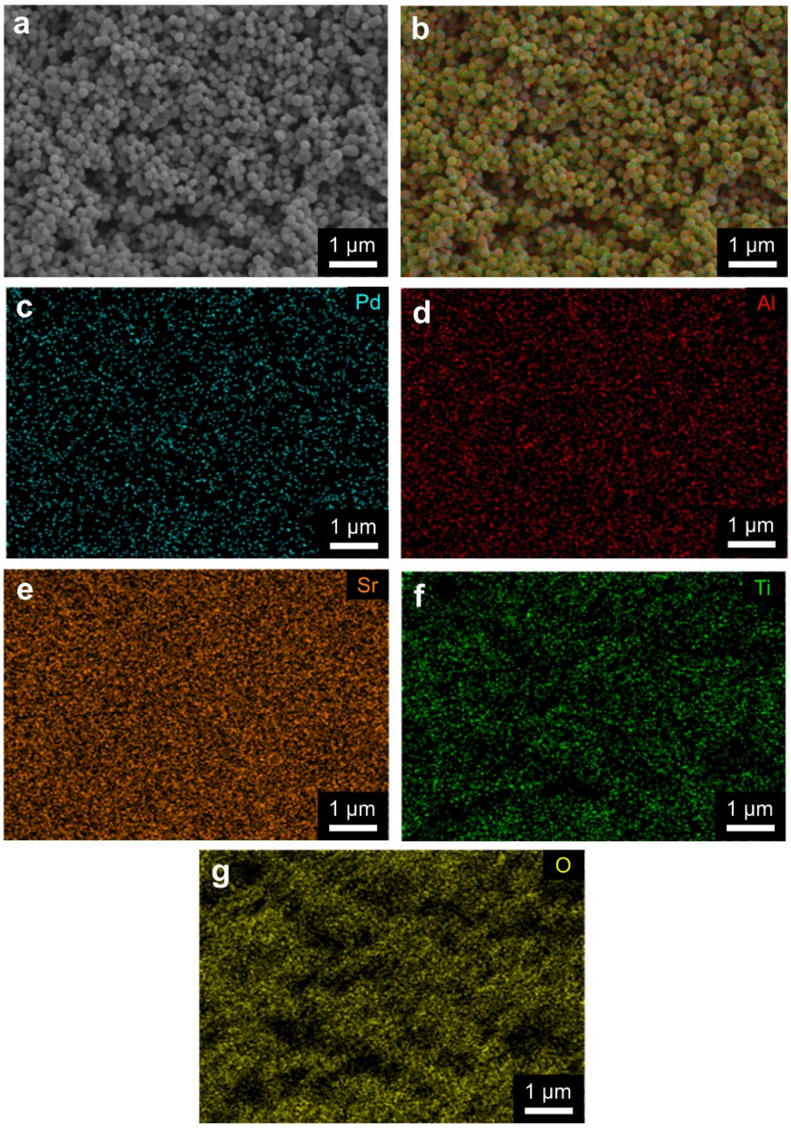

Fig. S4. Top-view SEM images and SEM-EDX elemental mapping of as-prepared Al:$SrTiO_3$|Pd_{CR} PC sheet. a, Top-view SEM image. **b-g,** Corresponding overall (**b**), Pd (**c**), Al (**d**), Sr (**e**), Ti (**f**) and O (**g**) EDX elemental mapping.

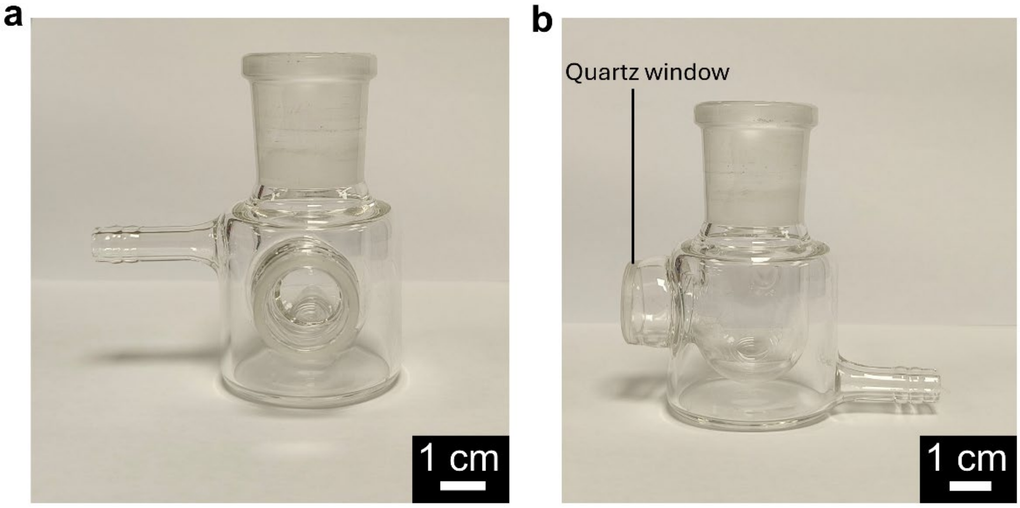

Fig. S5. Photographs of the side-irradiation glass photoreactor used for small-scale (1 cm^2) photocatalytic reactions. a,b, Front-view (**a**) and side-view (**b**) photographs of the photoreactor.

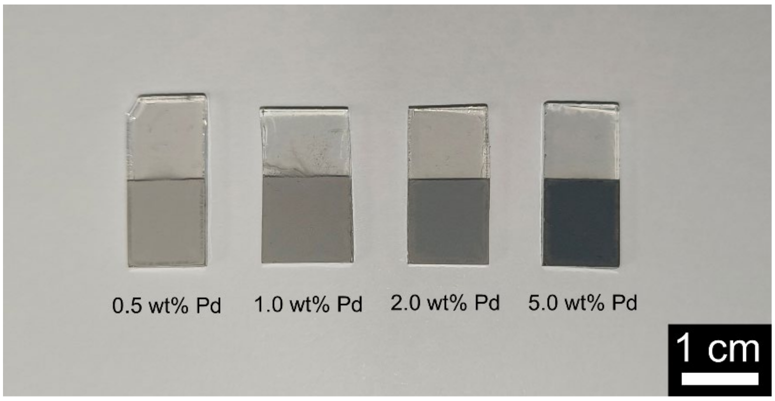

Fig. S6. Photographs of Al:$SrTiO_3$|Pd_{CR} PC sheets with Pd loadings from 0.5−5.0 wt%. It can be seen that the colour of the PC sheet becomes much darker with increasing Pd loadings, thus hindering light absorption at high Pd loadings.

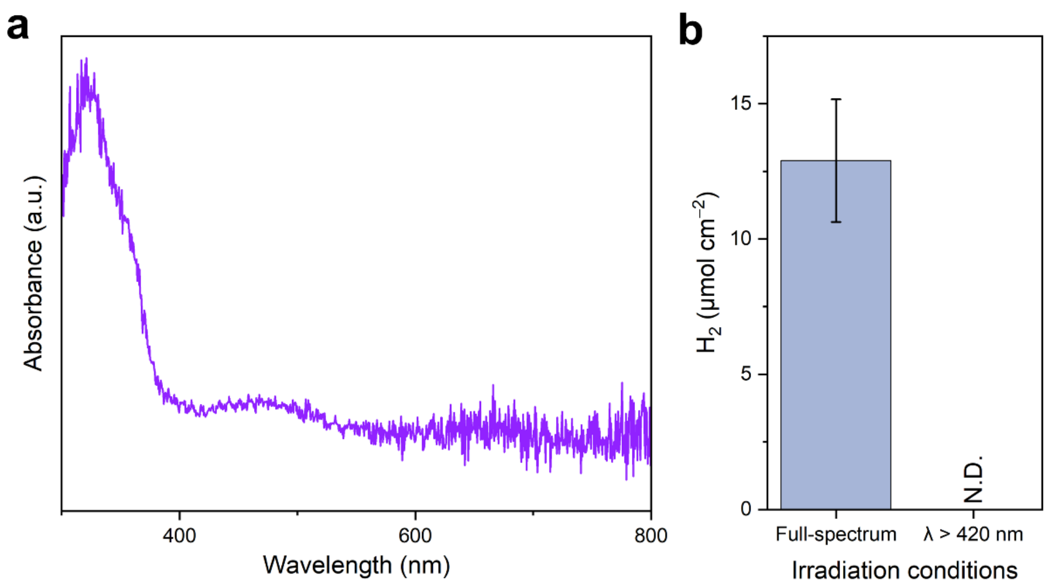

Fig. S7. Control experiments to study the possibility of plasmonic light absorption by Pd cocatalyst. a, UV−Vis diffuse reflectance spectra of Al:$SrTiO_3$|Pd_{CR}. **b,** H_2 evolution from the Al:$SrTiO_3$|Pd_{CR} PC sheets under full-spectrum and λ > 420 nm irradiation. The photocatalytic experiments were performed in 1.0 M KOH (pH 14) containing 0.1 M EG for 24 h at 25 °C with stirring.

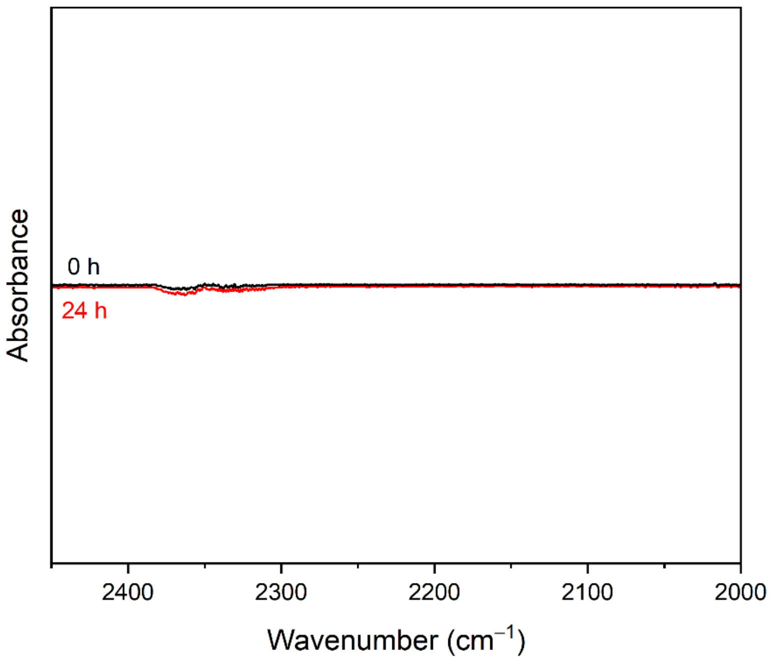

Fig. S8. IR spectra of headspace gas before and after EG photoreforming experiment using Al:$SrTiO_3$|Pd_{CR} PC sheet. Absorption peaks of CO_2 and CO are expected to be at ~2350 and ~2150 cm^{-1}, respectively. After withdrawing the headspace gas for the 0 h measurement, the reactor was re-purged with N_2 (with 2% CH_4 as internal standard) and the experiment was performed as normal. The photocatalytic experiment was performed under AM1.5G illumination for 24 h at 25 °C with stirring.

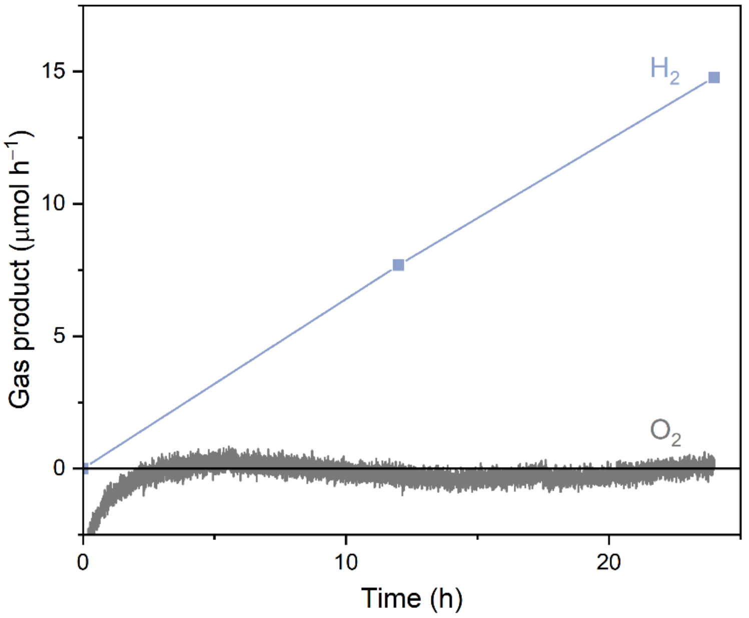

Fig. S9. Time-course H_2 and O_2 evolution from Al:$SrTiO_3$|Pd_{CR} PC sheet during EG photoreforming. The data shows that no O_2 had evolved from the experiment, indicating that water oxidation as a side reaction did not occur. The photocatalytic experiment was performed under AM1.5G illumination for 24 h at 25 °C with stirring.

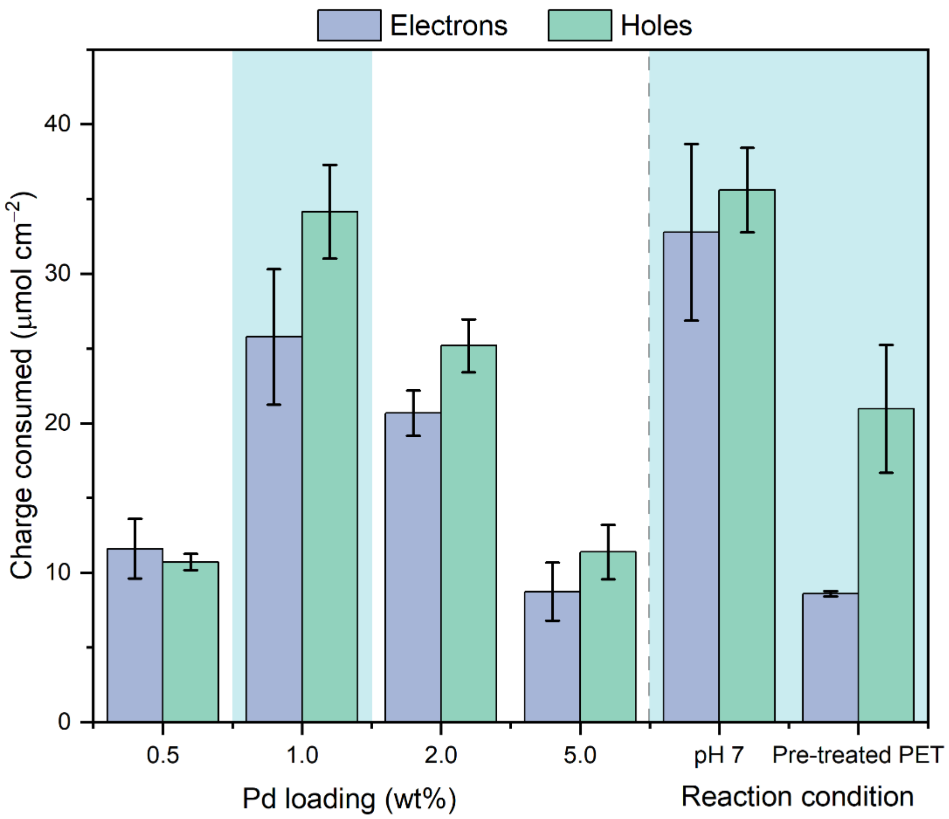

Fig. S10. Charge consumed in the oxidation and reduction reaction in experiments on Al:$SrTiO_3$|Pd_{CR} PC sheets with different Pd loadings, at different pH and using pre-treated waste commercial PET bottles as the oxidation substrate. Data highlighted in light blue indicate the optimal Pd loading that was subsequently used in experiments with varying reaction condition. The photocatalytic experiments were performed under AM1.5G illumination for 24 h at 25 °C with stirring.

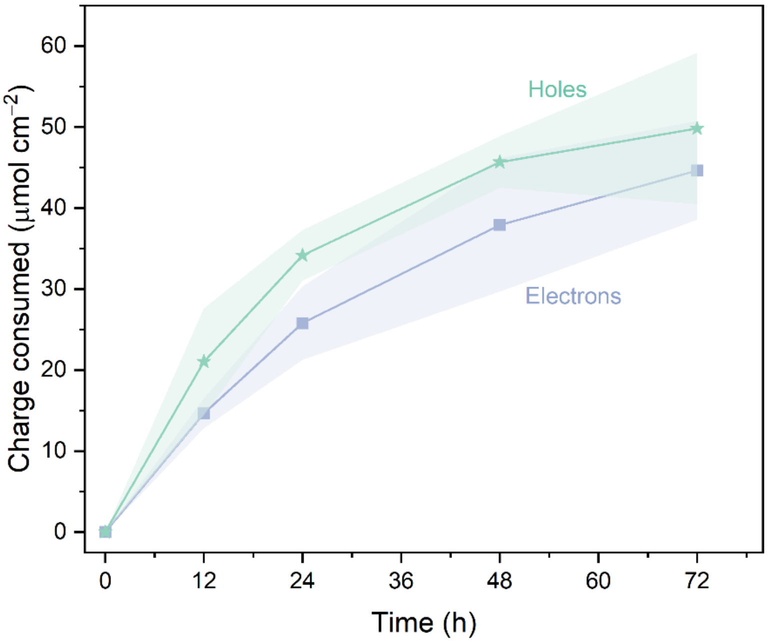

Fig. S11. Charge consumed in the oxidation and reduction reaction in experiments on long-term performance of Al:$SrTiO_3$|Pd_{CR} PC sheets. Photocatalytic experiments were performed under AM1.5G illumination for 72 h at 25 °C with stirring.

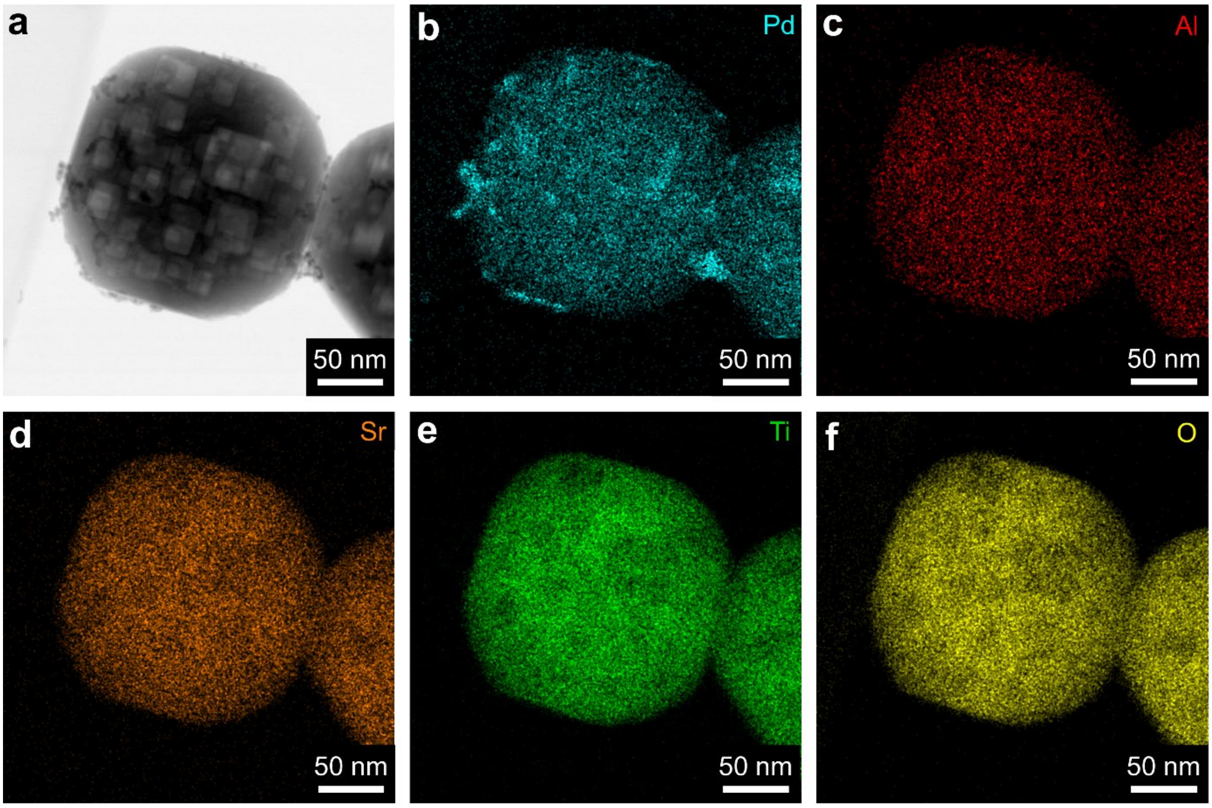

Fig. S12. BF-STEM image and STEM-EDX elemental mapping of Al:$SrTiO_3$|Pd_{CR} after catalysis. a, Bright-field STEM image of Al:$SrTiO_3$|Pd_{CR} powder after catalysis. **b-f,** Corresponding Pd (**b**), Al (**c**), Sr (**d**), Ti (**e**) and O (**f**) EDX elemental mapping. No Pd agglomeration was observed after catalysis.

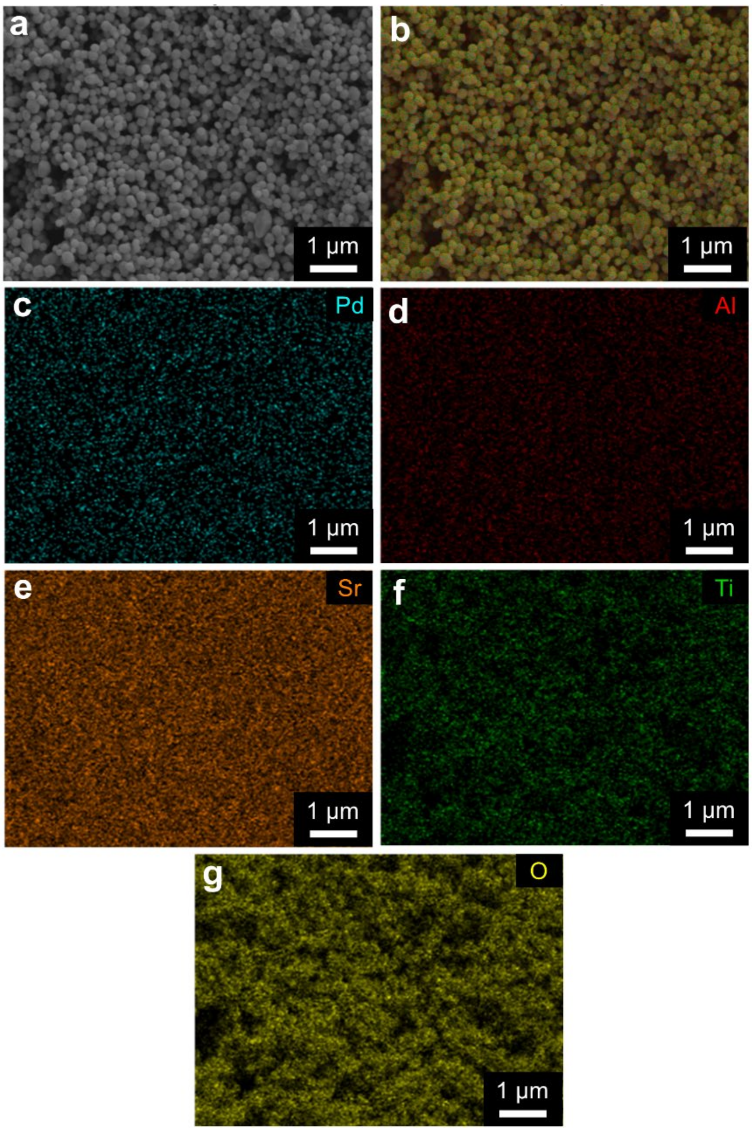

Fig. S13. Top-view SEM images and SEM-EDX elemental mapping of Al:$SrTiO_3$|Pd_{CR} PC sheet after catalysis. a, Top-view SEM image. **b-g,** Corresponding overall (**b**), Pd (**c**), Al (**d**), Sr (**e**), Ti (**f**) and O (**g**) EDX elemental mapping.

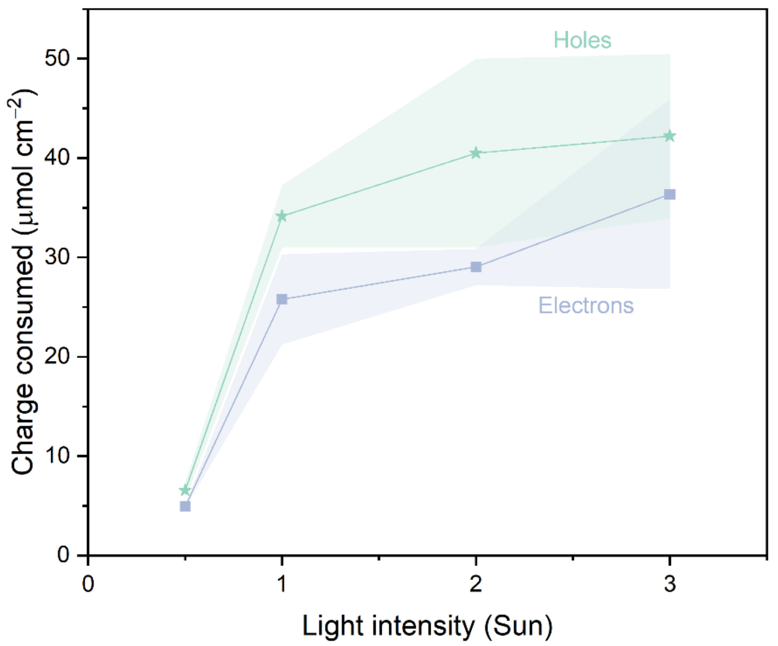

Fig. S14. Charge consumed in the oxidation and reduction reaction in experiments on Al:$SrTiO_3$|Pd_{CR} PC sheets under different light intensities. Photocatalytic experiments were performed under AM1.5G illumination for 24 h at 25 °C with stirring.

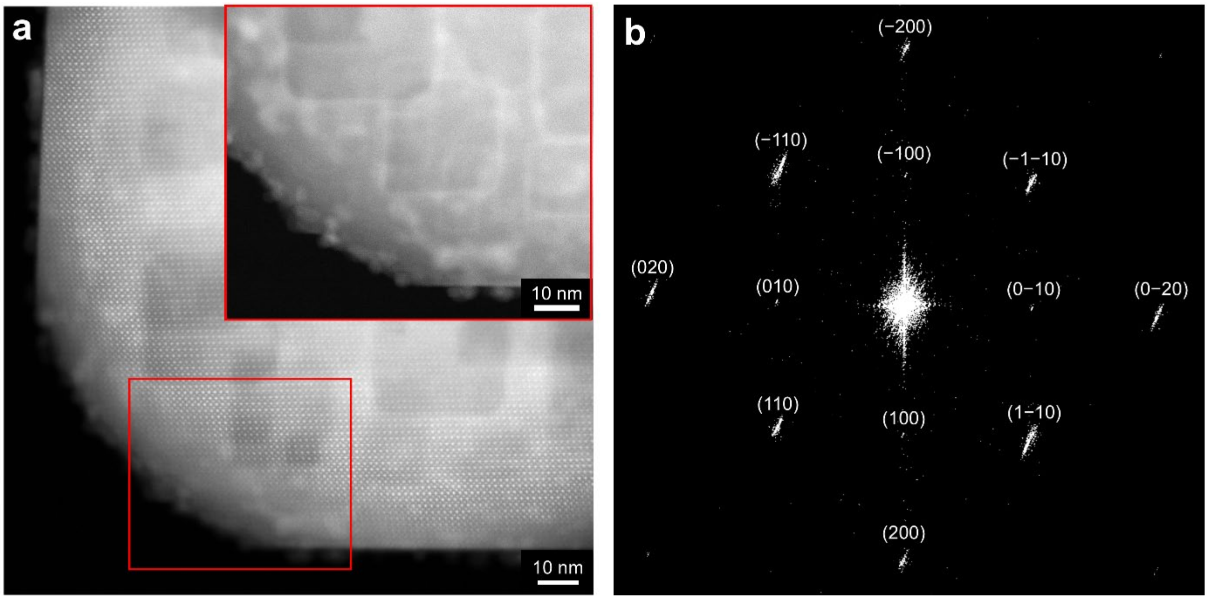

Fig. S15. HAADF-STEM image of Al:$SrTiO_3$|Pd_{CR} powder along the [001] zone axis and corresponding FFT pattern. a, HAADF-STEM image of Al:$SrTiO_3$|Pd_{CR} powder. Inset: magnified view of the region bounded by the red square. **b,** FFT pattern obtained from (**a**).

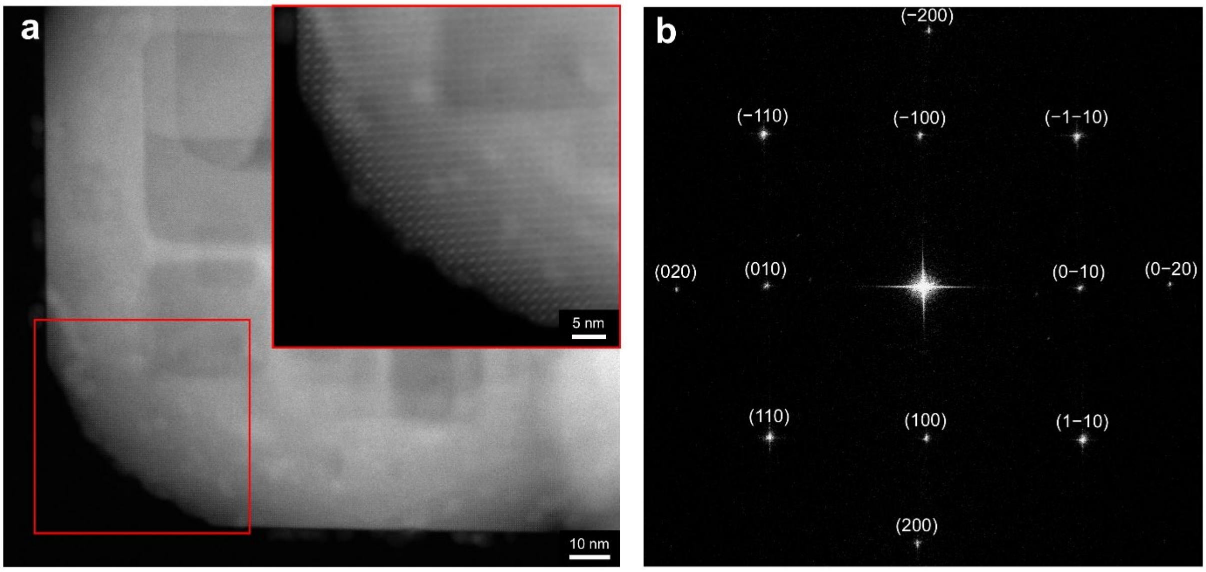

Fig. S16. HAADF-STEM image of Al:$SrTiO_3$|Pd_{PR} powder along the [001] zone axis and corresponding FFT pattern. a, HAADF-STEM image of Al:$SrTiO_3$|Pd_{PR} powder. Inset: magnified view of the region bounded by the red square. **b,** FFT pattern obtained from (**a**).

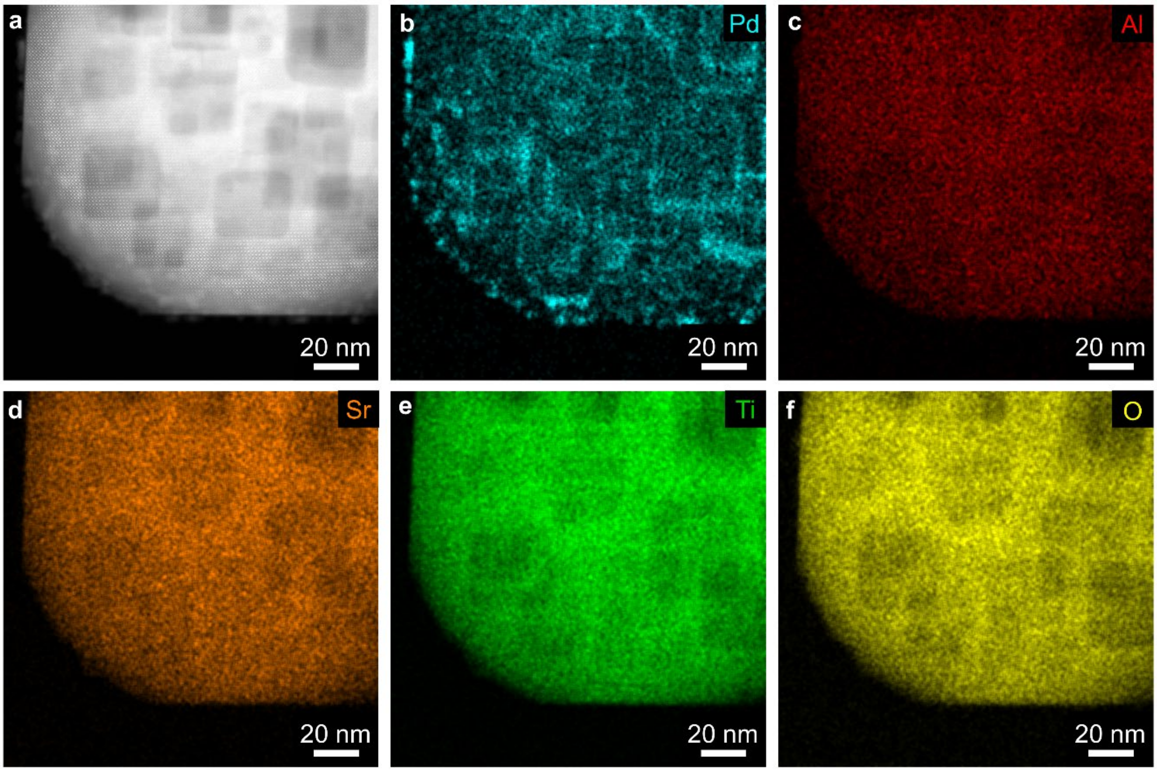

Fig. S17. HAADF-STEM images and EDX elemental mapping of Al:$SrTiO_3$|Pd_{CR} powder along the [001] zone axis. a, HAADF-STEM image of Al:$SrTiO_3$|Pd_{CR} powder. **b-f** Corresponding Pd (**b**), Al (**c**), Sr (**d**), Ti (**e**) and O (**f**) EDX elemental mapping.

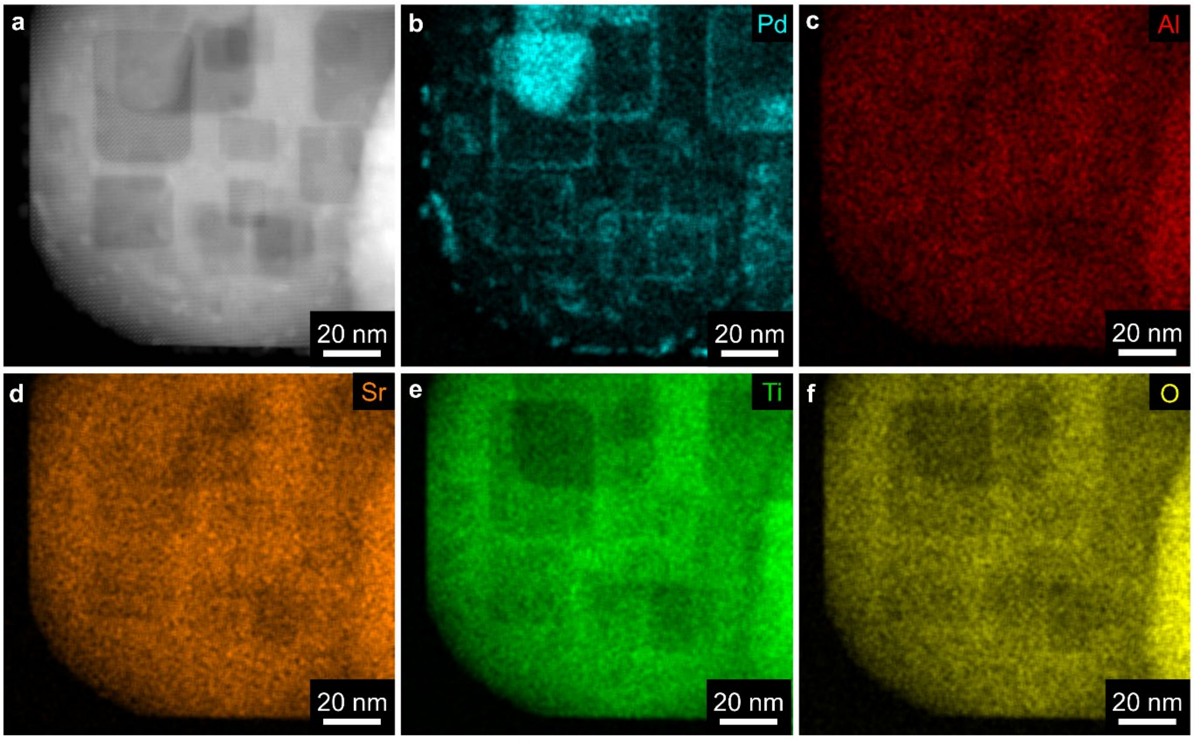

Fig. S18. HAADF-STEM images and EDX elemental mapping of Al:SrTiO3|PdPR powder along the [001] zone axis. a, HAADF-STEM image of Al:SrTiO3|PdPR powder. **b-f** Corresponding Pd (**b**), Al (**c**), Sr (**d**), Ti (**e**) and O (**f**) EDX elemental mapping.

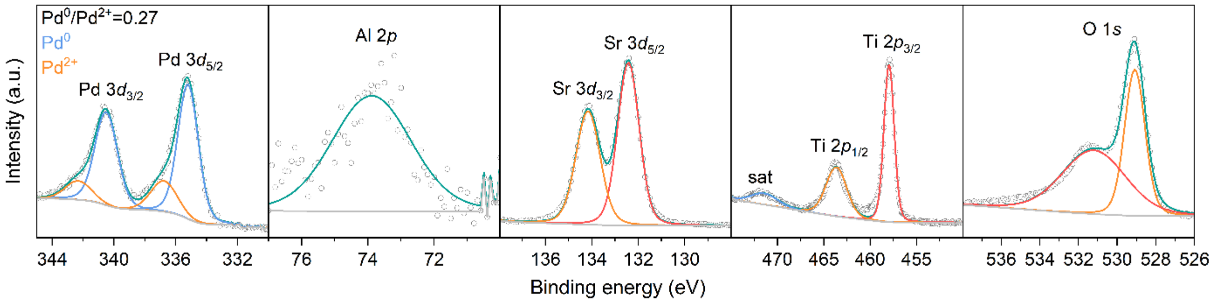

Fig. S19. Curve-fitted XPS spectra of as-prepared Al:SrTiO3|PdPR PC sheets. The spectra show that Pd species were present in the 0 and 2+ oxidation states (due to PdO formation from aerial oxidation) and that each of the other expected elements were present. Sat., satellite peak.

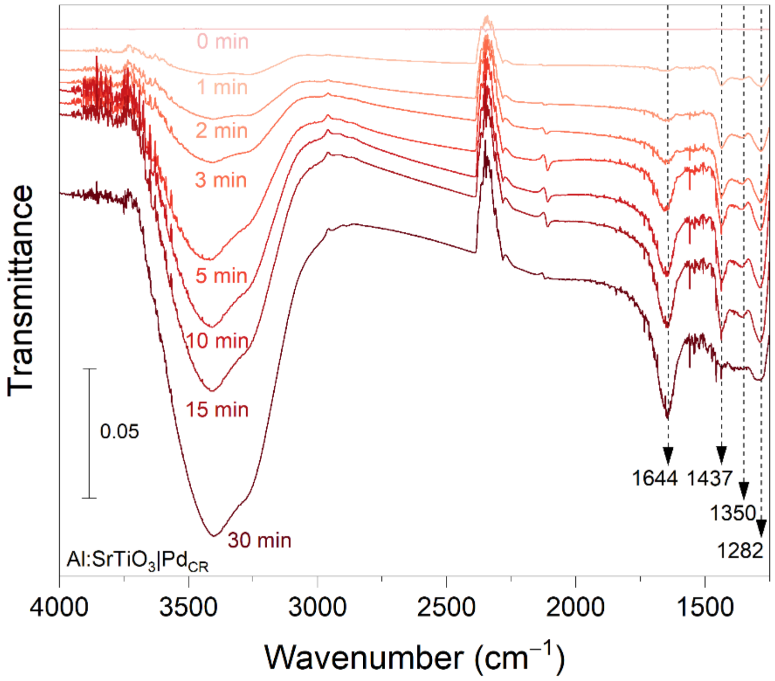

Fig. S20. Full in situ ATR-IR spectra of EG photoreforming by Al:$SrTiO_3$|Pd_{CR} at pH 14. All spectra have been background-subtracted. The background and t=0 min spectra were collected in the dark, after which light illumination was started to begin the experiment. The same spectrum with truncated wavenumber axis is shown in Figure 3d.

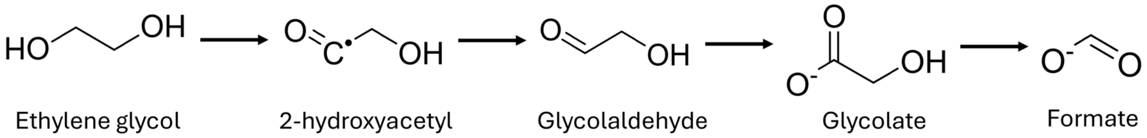

Fig. S21. Schematic illustration of proposed reaction pathway of EG oxidation. Glycolaldehyde self-dimerises under reaction conditions to form GAld dimer.[34]

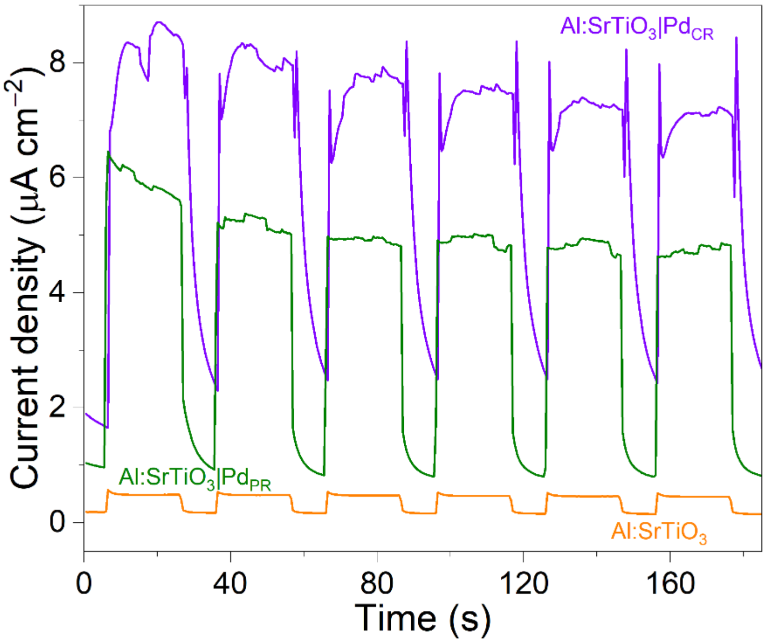

Fig. S22. Chopped light chronoamperometry traces of Al:$SrTiO_3$|Pd_{CR}, Al:$SrTiO_3$|Pd_{PR} and bare Al:$SrTiO_3$. PEC measurements were performed in 30 ml stirred, N_2-saturated 1.0 M KOH (pH 14) electrolyte containing 0.1 M EG under AM1.5G illumination. The observed photocurrent fluctuations are attributed to the constant stirring of the electrolyte to overcome mass transport limitations, rather than from catalyst detachment, as the latter case would result in continuous decay in the overall photocurrent.[35]

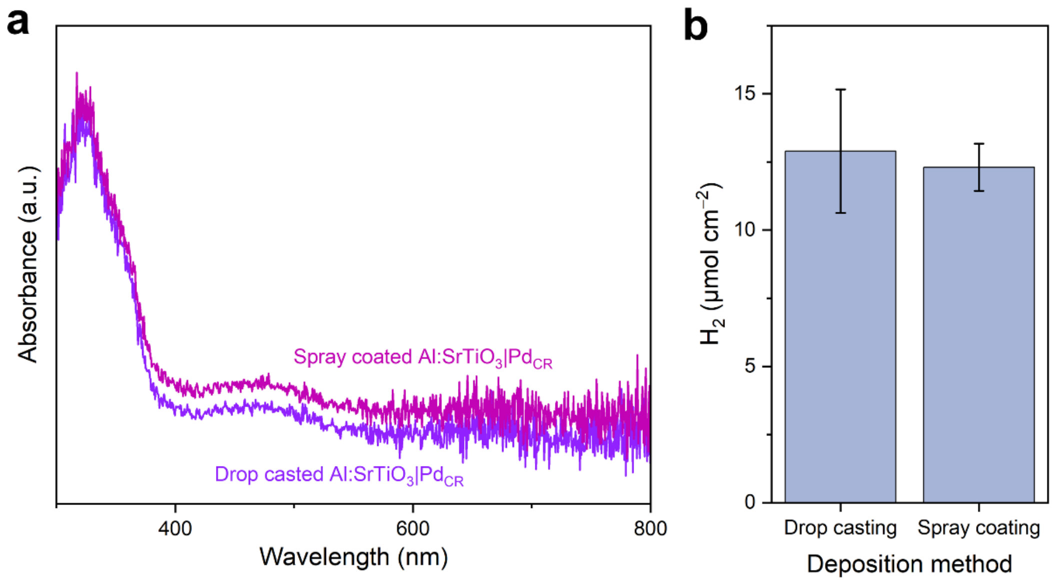

Fig. S23. Performance comparison between small-scale PC sheet fabrication by drop casting and spray coating Al:SrTiO$_3$|Pd$_{CR}$. a, UV−Vis diffuse reflectance spectra of Al:SrTiO$_3$|Pd$_{CR}$ PC sheets fabricated by drop casting and spray coating. **b,** H_2 evolution from the Al:SrTiO$_3$|Pd$_{CR}$ PC fabricated by drop casting and spray coating. The photocatalytic experiments were performed in 1.0 M KOH (pH 14) containing 0.1 M EG under AM1.5G illumination for 24 h at 25 °C with stirring.

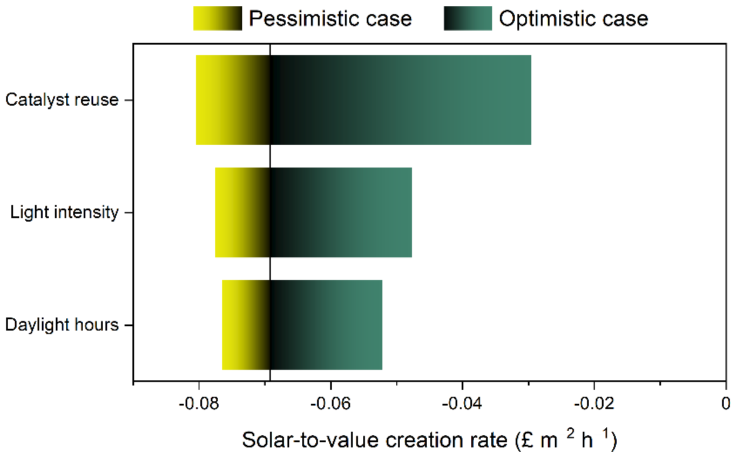

Fig. S24. Sensitivity analysis of STV creation rate considering only photocatalytic aspects (i.e., without considering waste PET pre-treatment) and excluding capital costs. Capital costs account for a large portion of the total cost of the solar reforming system, highlighting the potential of economic incentives by governing bodies in pushing emerging solar reforming technologies towards profitability.

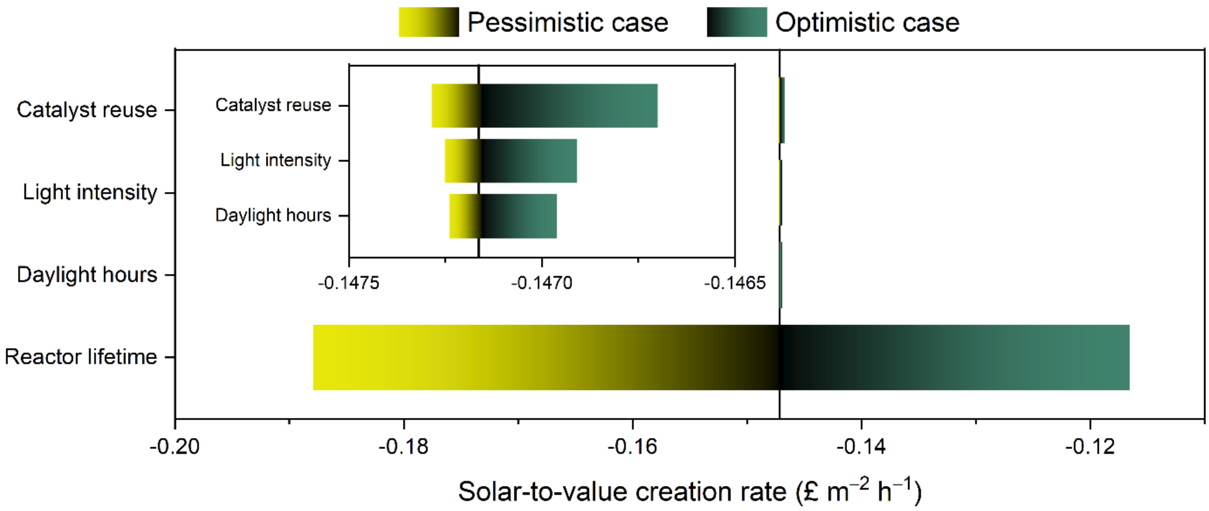

Fig. S25. Sensitivity analysis of the STV creation rate of a hypothetical overall water splitting system. Inset: zoomed-in view of the data exluding reactor lifetime.

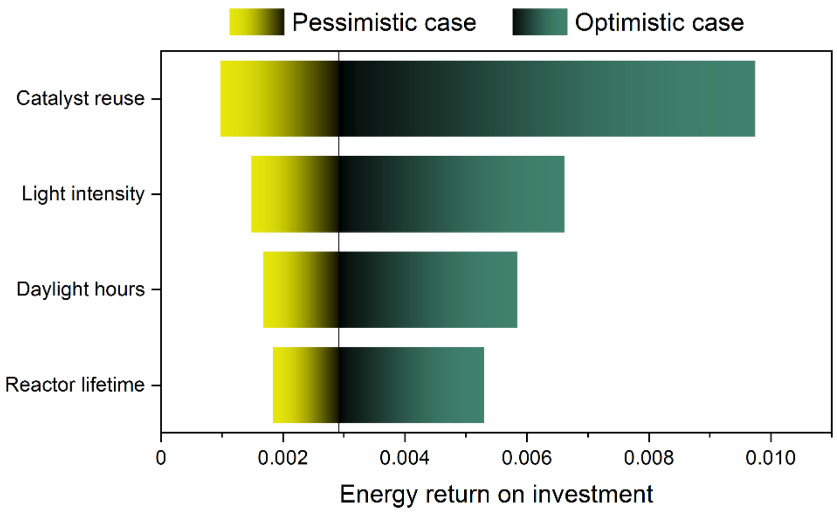

Fig. S26. Sensitivity analysis of the EROI of a hypothetical overall water splitting system. The EROI of the hypothetical overall water splitting system is much lower than that of the PET reforming system due to O_2 production instead of valorised organics production.

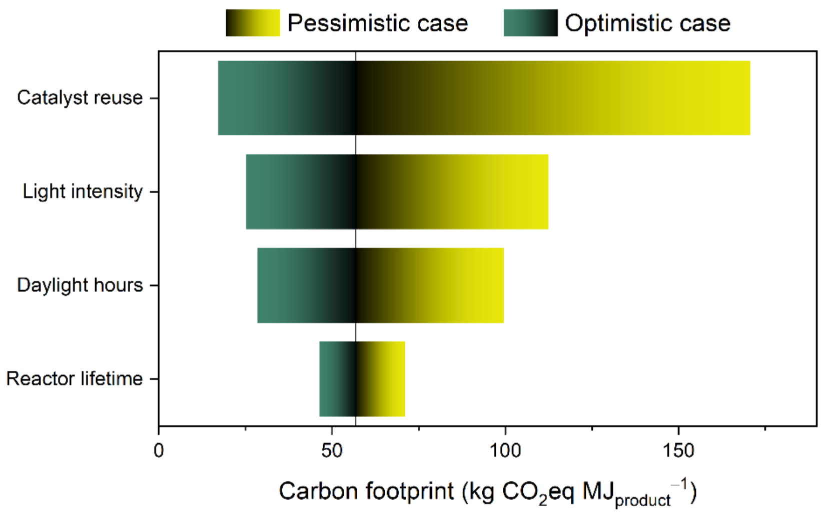

Fig. S27. Sensitivity analysis of the carbon footprint of a hypothetical overall water splitting system. The carbon footprint of the hypothetical overall water splitting system is much lower than that of the PET reforming system due to the lack of waste upcycling aspects in the former system.

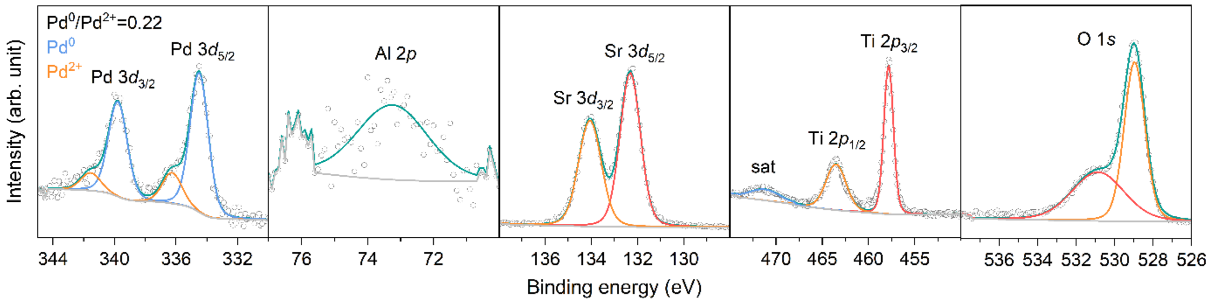

Fig. S28. Curve-fitted XPS spectra of post-catalysis Al:$SrTiO_3$|Pd_{CR} PC sheets. The increase in Pd^0/Pd^{2+} ratio after catalysis compared to before catalysis (Fig. 1i) indicates that some of the electrons generated were consumed for the partial reduction of Pd over the course of the photocatalytic reactions. Sat., satellite peak.

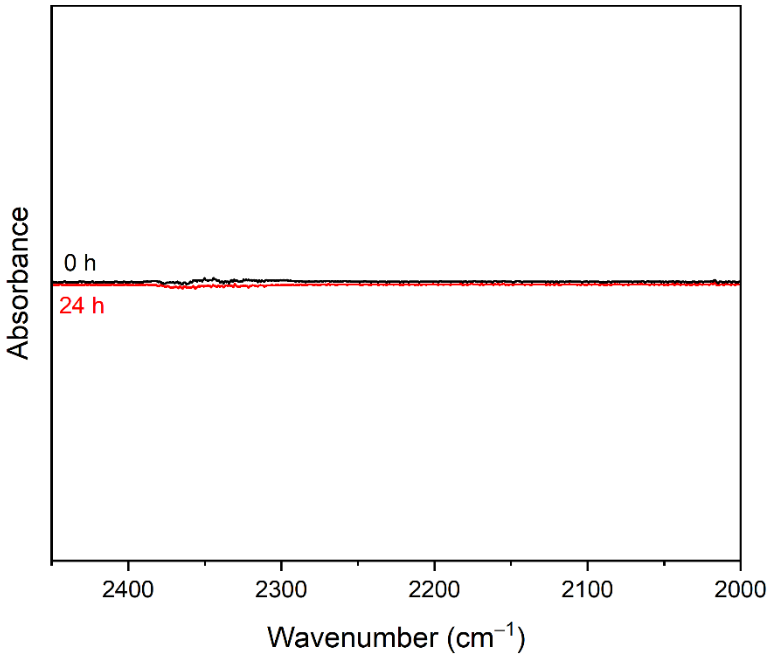

Fig. S29. IR spectra of headspace gas before and after EG photoreforming experiment using Al:$SrTiO_3$|Pd_{PR} PC sheet. Absorption peaks of CO_2 and CO are expected to be at ~2350 and ~2150 cm^{-1}, respectively. After withdrawing the headspace gas for the 0 h measurement, the reactor was re-purged with N_2 (with 2% CH_4 as internal standard) and the experiment was performed as normal. The photocatalytic experiment was performed under AM1.5G illumination for 24 h at 25 °C with stirring.

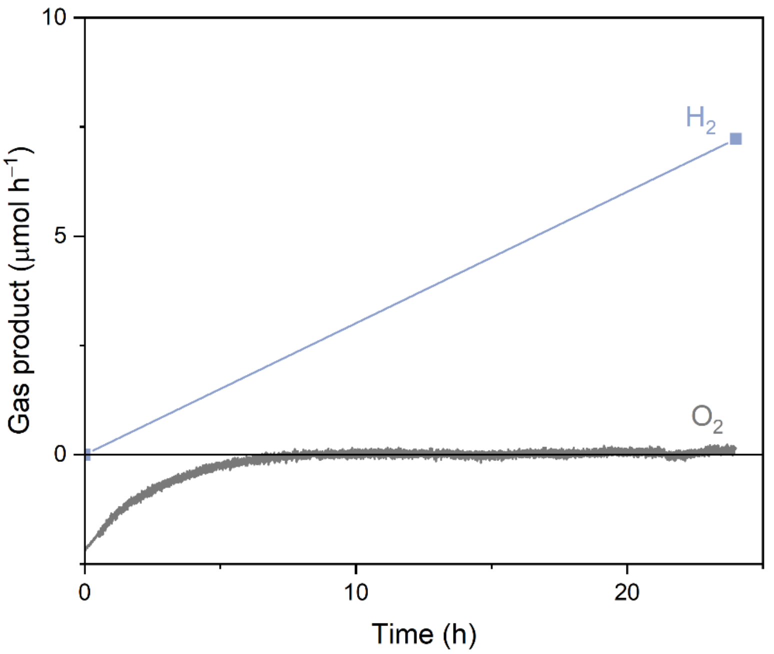

Fig. S30. Time-course H_2 and O_2 evolution from Al:SrTiO$_3$|Pd$_{PR}$ PC sheet during EG photoreforming. The data shows that no O_2 had evolved from the experiment, indicating that water oxidation as a side reaction did not occur. The photocatalytic experiment was performed under AM1.5G illumination for 24 h at 25 °C with stirring.

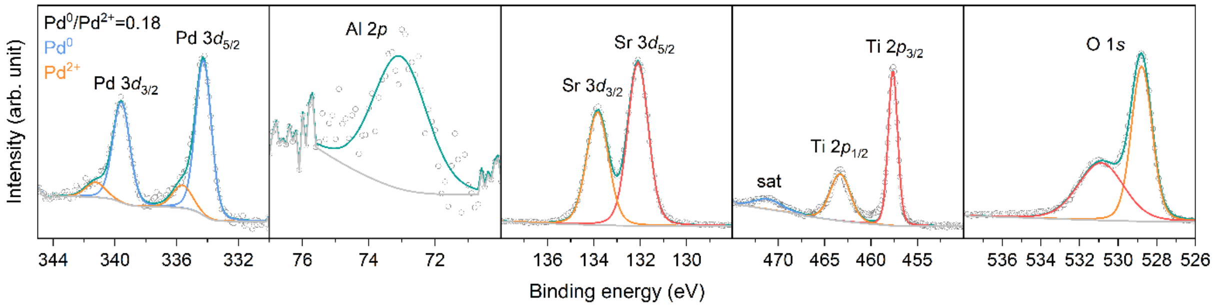

Fig. S31. Curve-fitted XPS spectra of post-catalysis Al:SrTiO$_3$|Pd$_{PR}$ PC sheets. The decrease in Pd^0/Pd^{2+} ratio after catalysis compared to before catalysis (Fig. S19) indicates that there was partial oxidation of Pd over the course of the photocatalytic reactions. Sat., satellite peak.

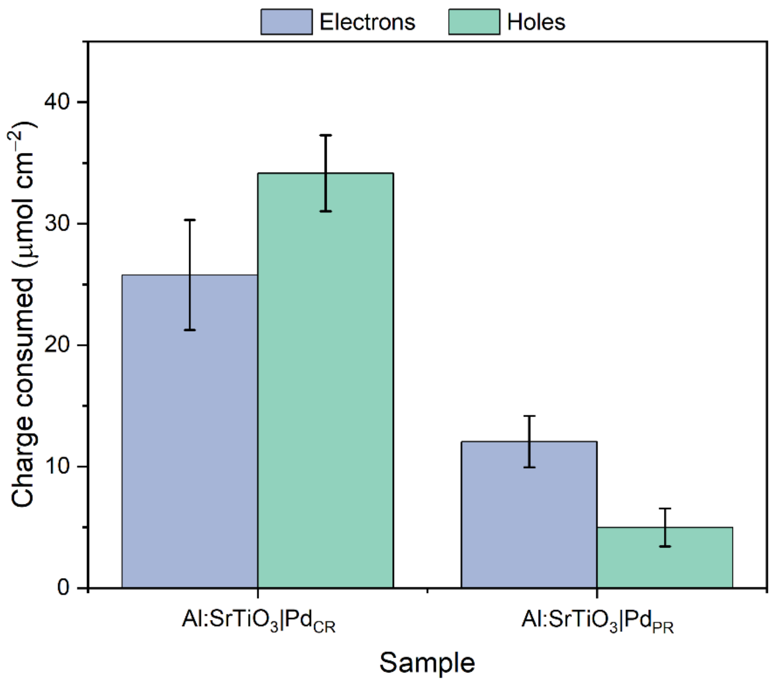

Fig. S32. Charge consumed in the oxidation and reduction reaction in experiments on Al:SrTiO$_3$|Pd$_{CR}$ and Al:SrTiO$_3$|Pd$_{PR}$ PC sheets. Photocatalytic experiments were performed under AM1.5G illumination for 24 h at 25 °C with stirring.

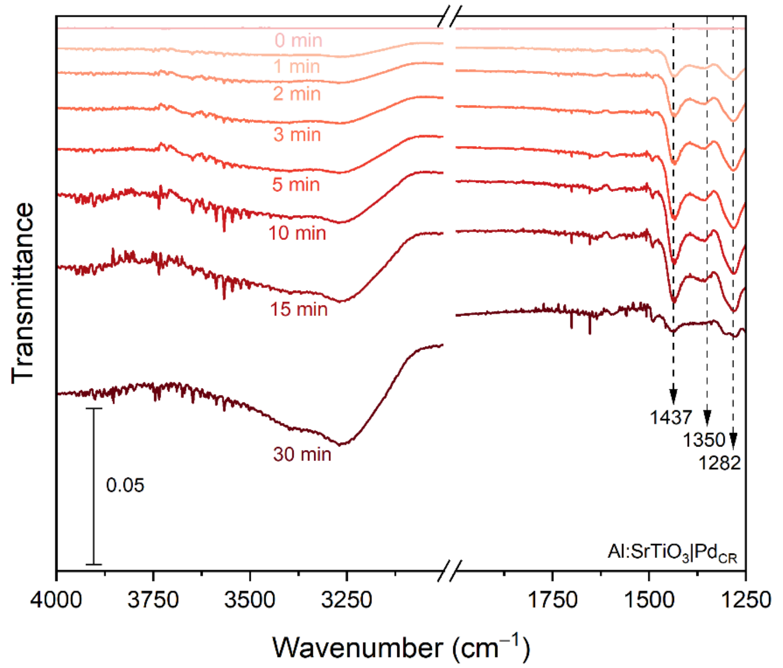

Fig. S33. In situ ATR-IR spectra of photocatalytic reforming of EG by Al:SrTiO$_3$|Pd$_{CR}$ at pH 7. All spectra have been background-subtracted. The background and t=0 min spectra were collected in the dark, after which light illumination was started to begin the experiment. The difference in band formation explains the difference in product formation at pH 14 versus pH 7 (Note S3).

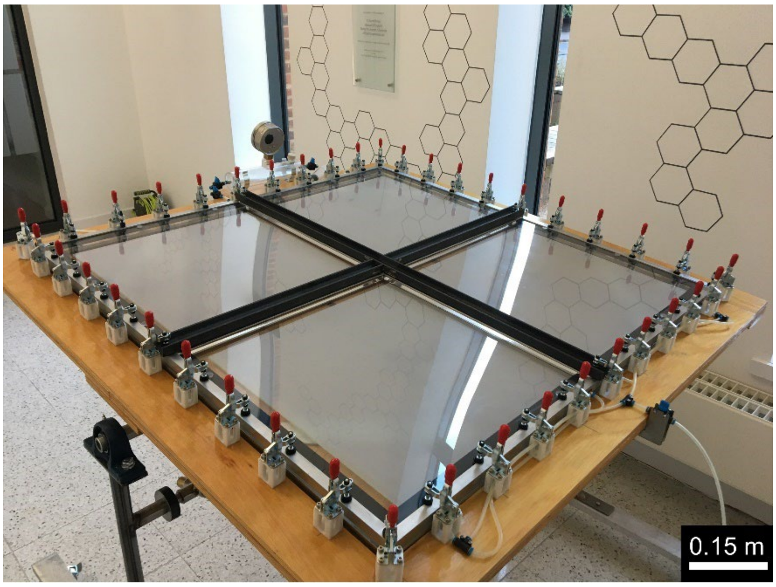

Fig. S34. Photograph of large-scale panel photoreactor. The photograph was taken indoors (Yusuf Hamied Department of Chemistry) before filling the reactor with pre-treated PET solution for the outdoor demonstration.

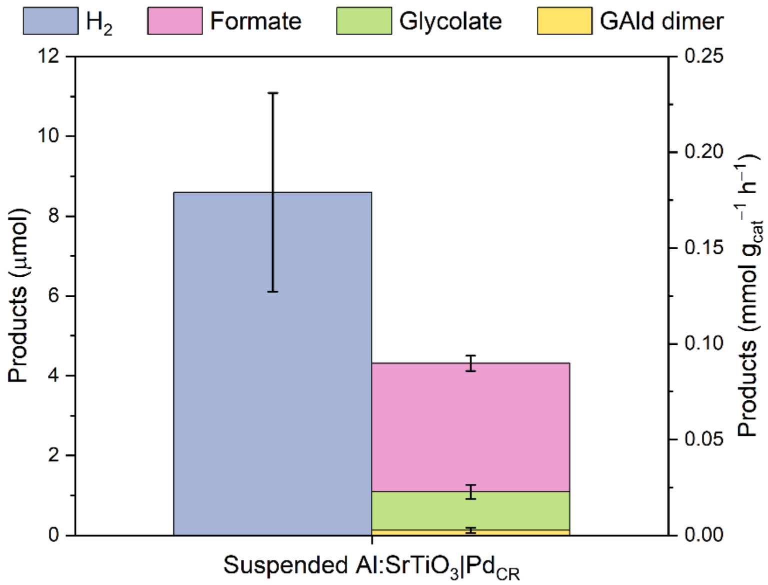

Fig. S35. Performance of Al:$SrTiO_3$|Pd_{CR} PC powder in a suspended system. The data is plotted in absolute amounts and on a per gram catalyst basis to facilitate comparison with other reports. The photocatalytic experiments were performed under AM1.5G illumination for 24 h at 25 °C with stirring.

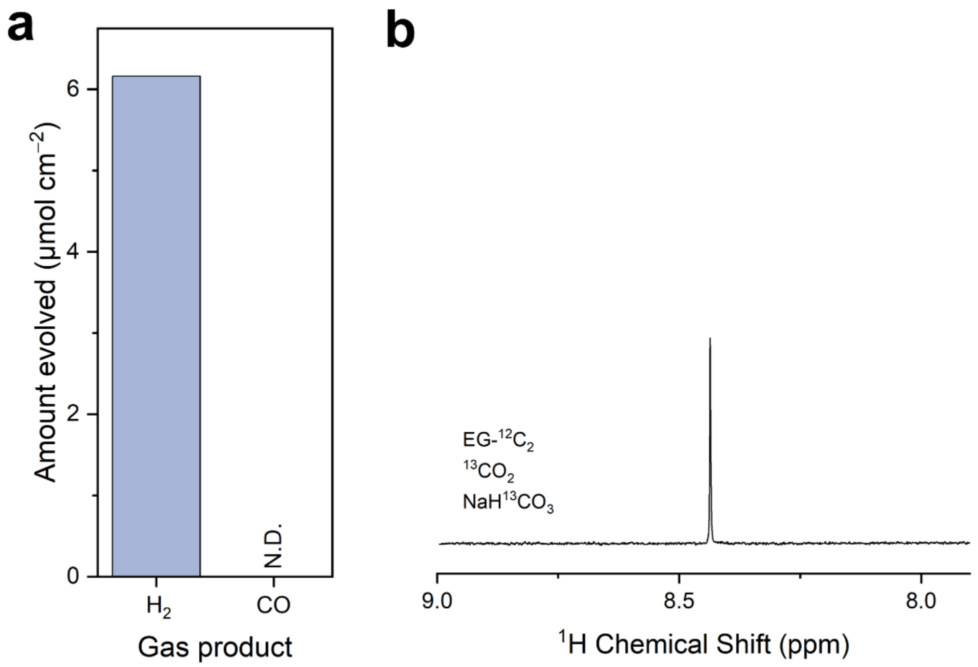

Fig. S36. CO_2 reduction test using the Al:$SrTiO_3$|Pd_{CR} PC sheet. a, Gas product evolution from the photocatalytic experiment. **b,** Formate detection by ^{1}H-NMR spectroscopy of the reaction solution after photocatalysis. The absence of formate-^{13}C is indicated by a lack of $J_{C\text{-}H}$ coupling induced doublet of formate. The photocatalytic experiment was performed in 0.1 M NaH^{13}CO$_3$ containing 0.1 M EG purged with ^{13}CO$_2$ under AM1.5G illumination for 24 h at 25 °C with stirring.

Table S1. Calculated and measured loadings of Pd on Al:$SrTiO_3$|Pd_{CR}. The calculated values were based on the amount of Pd precursor used during Al:$SrTiO_3$|Pd_{CR} synthesis and the measured values were determined via ICP-OES.

Calculated as-prepared Pd loading (wt%)	Measured Pd loading (wt%)	
	As-prepared	Post-catalysis
0.5	0.46	0.46
1.0	0.91	0.91
2.0	1.80	1.80
5.0	4.34	4.34
1.0 (photoreduced)	0.86	0.86

Table S2. Performance of Al:$SrTiO_3$|Pd_{CR} PC sheets with different Pd loadings. The photocatalytic experiments were performed under AM1.5G illumination for 24 h at 25 °C with stirring.

Pd loading (wt%)	H_2 (μmol cm^{-2})	Formate (μmol cm^{-2})	Glycolate (μmol cm^{-2})	GAld dimer (μmol cm^{-2})	Calculated H_2 (μmol cm^{-2})
0.5	5.8±1.0	1.8±0.2	1.3±0.2	0.1±0.1	5.4±0.3
1.0	12.9±2.3	9.6±1.7	1.1±0.5	0.5±0.3	17.1±1.6
2.0	10.3±0.8	7.4±0.3	0.8±0.2	0.02±0.02	12.6±0.9
5.0	4.4±1.0	2.7±0.6	0.8±0.0004	0.01±0.02	5.7±0.9

Table S3. Exclusion control experiments showing H_2 evolution under standard conditions and with certain components of the photoreforming system removed. The photocatalytic experiments were performed under AM1.5G illumination for 24 h at 25 °C with stirring. N.D., not detected.

Reaction condition				H_2 (μmol cm^{-2})
EG	Pd cocatalyst	Light absorber	Light	
✓	✓	✓	✓	12.9±2.3
✗	✓	✓	✓	0.1±0.001
✓	✗	✓	✓	0.2±0.04
✓	✗	✗	✓	N.D.
✓	✓	✓	✗	N.D.

Table S4. Charge consumed in the oxidation and reduction reaction in experiments on Al:SrTiO$_3$|Pd$_{CR}$ PC sheets with different Pd loadings. The photocatalytic experiments were performed under AM1.5G illumination for 24 h at 25 °C with stirring.

Pd loading (wt%)	Charged consumed (μmol cm^{-2})	
	Electrons	Holes
0.5	11.6±2.0	10.7±0.5
1.0	25.8±4.5	34.2±3.1
2.0	20.7±1.5	25.2±1.8
5.0	8.7±2.0	11.4±1.8

Table S5. Time-course product evolution from long-term experiments using Al:SrTiO$_3$|Pd$_{CR}$ PC sheets. Photocatalytic experiments were performed under AM1.5G illumination for 72 h at 25 °C with stirring.

Time (h)	H_2 (μmol cm^{-2})	Formate (μmol cm^{-2})	Glycolate (μmol cm^{-2})	GAld dimer (μmol cm^{-2})	Calculated H_2 (μmol cm^{-2})
0	0	0	0	0	0
12	7.3±0.9	6.0±2.4	0.8±0.2	0	10.5±3.3
24	12.9±2.3	9.6±1.7	1.1±0.5	0.5±0.3	17.1±1.6
48	19.0±4.1	11.8±1.5	2.1±0.3	0.9±0.7	22.8±1.6
72	22.3±3.1	13.0±3.3	2.2±0.3	1.0±0.5	24.9±4.7

Table S6. Charge consumed in the oxidation and reduction reaction in experiments on long-term performance of Al:SrTiO$_3$|Pd$_{CR}$ PC sheets. Photocatalytic experiments were performed under AM1.5G illumination for 72 h at 25 °C with stirring.

Time (h)	Charged consumed (μmol cm^{-2})	
	Electrons	Holes
0	0	0
12	14.6±1.8	21.0±6.6
24	25.8±4.5	34.2±3.1
48	37.9±8.2	45.7±3.2
72	44.6±6.1	49.8±9.3

Table S7. Performance of Al:SrTiO$_3$|Pd$_{CR}$ PC sheets at different pH. The photocatalytic experiments were performed under AM1.5G illumination for 24 h at 25 °C with stirring.

pH	H_2 (μmol cm^{-2})	Formate (μmol cm^{-2})	Glycolate (μmol cm^{-2})	GAld dimer (μmol cm^{-2})	Calculated H_2 (μmol cm^{-2})
14	12.9±2.3	9.6±1.7	1.1±0.5	0.5±0.3	17.1±1.6
7	16.4±3.0	6.4±1.4	1.0±0.5	6.2±0.7	17.8±1.4

Table S8. Performance of Al:SrTiO$_3$|Pd$_{CR}$ PC sheets at different light intensities. The photocatalytic experiments were performed under AM1.5G illumination for 24 h at 25 °C with stirring.

Light intensity (Sun)	H_2 (μmol cm^{-2})	Formate (μmol cm^{-2})	Glycolate (μmol cm^{-2})	GAld dimer (μmol cm^{-2})	Calculated H_2 (μmol cm^{-2})
0.5	2.5±0.1	1.0±0.2	0.7±0.2	0.4±0.2	3.3±0.4
1.0	12.9±2.3	9.6±1.7	1.1±0.5	0.5±0.3	17.1±1.6
2.0	14.5±0.9	8.7±2.2	3.2±1.0	0.8±0.5	20.3±4.8
3.0	18.2±4.8	9.1±2.0	3.6±0.7	0.3±0.2	21.1±4.1

Table S9. Performance of Al:SrTiO$_3$|Pd$_{CR}$ PC sheets using EG and pre-treated waste commercial PET bottles as the oxidation substrate. The photocatalytic experiments were performed under AM1.5G illumination for 24 h at 25 °C with stirring.

Substrate	H_2 (μmol cm^{-2})	Formate (μmol cm^{-2})	Glycolate (μmol cm^{-2})	GAld dimer (μmol cm^{-2})	Calculated H_2 (μmol cm^{-2})
EG	12.9±2.3	9.6±1.7	1.1±0.5	0.5±0.3	17.1±1.6
Pre-treated PET	4.3±0.1	4.6±1.2	1.6±0.2	0.3±0.1	10.5±2.1

Table S10. Charge consumed in the oxidation and reduction reaction in experiments on Al:SrTiO$_3$|Pd$_{CR}$ PC sheets at different pH. The photocatalytic experiments were performed under AM1.5G illumination for 24 h at 25 °C with stirring.

pH	Charged consumed (μmol cm^{-2})	
	Electrons	Holes
14	25.8±4.5	34.2±3.1
7	32.8±5.9	35.6±2.8

Table S11. Charge consumed in the oxidation and reduction reaction in experiments on Al:SrTiO$_3$|Pd$_{CR}$ PC sheets under different light intensities. Photocatalytic experiments were performed under AM1.5G illumination for 24 h at 25 °C with stirring.

Light intensity (Sun)	Charged consumed (μmol cm^{-2})	
	Electrons	Holes
0.5	5.0±0.1	6.5±0.9
1.0	25.8±4.5	34.2±3.1
2.0	29.0±1.8	40.5±9.5
3.0	36.4±9.6	42.2±8.3

Table S12. Charge consumed in the oxidation and reduction reaction in experiments on Al:$SrTiO_3$|Pd_{CR} PC sheets using EG and pre-treated waste commercial PET bottles as the oxidation substrate. Photocatalytic experiments were performed under AM1.5G illumination for 24 h at 25 °C with stirring.

Substrate	Charged consumed (μmol cm^{-2})	
	Electrons	Holes
EG	25.8±4.5	34.2±3.1
Pre-treated PET	8.6±0.2	21.0±4.3

Table S13. Product evolution from Al:$SrTiO_3$|Pd_{CR} and Al:$SrTiO_3$|Pd_{PR} PC sheets. Photocatalytic experiments were performed under AM1.5G illumination for 24 h at 25 °C with stirring.

Sample	H_2 (μmol cm^{-2})	Formate (μmol cm^{-2})	Glycolate (μmol cm^{-2})	GAld dimer (μmol cm^{-2})	Calculated H_2 (μmol cm^{-2})
Al:$SrTiO_3$|Pd_{CR}	12.9±2.3	9.6±1.7	1.1±0.5	0.5±0.3	17.1±1.6
Al:$SrTiO_3$|Pd_{PR}	6.0±1.1	0.4±0.2	0.9±0.2	0.1±0.1	2.5±0.8

Table S14. Wavenumbers observed by in situ ATR-IR spectroscopy and assigned species.

Species	Wavenumber (cm^{-1})	Ref
EG	1437, 1282	[36]
OH	3250−3500	[37]
Formate	1350	[38]
Glycolate	1350	[38]
2-hydroxyacetyl	1644	[18]

Table S15. PEIS fitting results. Error bars represent the fitting errors. PEC measurements were performed in 30 ml stirred, N_2-saturated 1.0 M KOH (pH 14) electrolyte containing 0.1 M EG under AM1.5G illumination.

Working electrode	R_{ct} (Ω cm^2)
Al:$SrTiO_3$|Pd_{CR}	535±6
Al:$SrTiO_3$|Pd_{PR}	1340±73
Al:$SrTiO_3$	2168±20

Table S16. Product formation over the course of the 1 m^2 outdoor demonstration. The photocatalytic experiments were performed under natural sunlight for three consecutive days (17-19 September 2024) for 7 h each day. Data shown is the cumulative amount of products generated over the course of the experiment.

Day	Time	H_2 (mmol m^{-2})	Formate (mmol m^{-2})	Glycolate (mmol m^{-2})	GAld dimer (mmol m^{-2})
17 September 2024	9:30 am	0	0	0	0
	1:30 pm	15.7	19.9	12.6	2.8
	16:30pm	22.6	29.5	12.4	2.4
18 September 2024	9:30 am	23.3	29.7	13.6	2.9
	1:30 pm	24.9	36.6	17.3	3.3
	16:30pm	36.9	34.7	14.5	2.8
19 September 2024	9:30 am	38.3	35.6	14.7	2.7
	1:30 pm	40.2	38.7	18.3	3.5
	16:30pm	48.1	44.9	18.5	2.6

Table S17. Reports on EG and PET photocatalytic reforming systems.

Photocatalytic system	Substrate	Light source	pH	Reaction scale (m^2 or g_{cat})[a]	Production rate ($mmol_{product}$ m^{-2} h^{-1} or $mmol_{product}$ g_{cat}^{-1} h^{-1})[b]		Stability (h)	Ref
					H_2	Oxidation products		
Al:SrTiO$_3$|Pd$_{CR}$	EG	Simulated sunlight; 100 mW cm^{-2}	14	0.002	0.18	Formate: 0.071<br>Glycolate: 0.021<br>GAld dimer: 0.0028	24	This work
			7	0.0001	6.83	Formate: 5.66<br>Glycolate: 3.00<br>GAld dimer: 2.58	24	
			14	0.0001	5.37	Formate: 4.65<br>Glycolate: 0.662<br>GAld dimer: 0.196	72	
	Pre-treated PET		14	0.0001	1.79	Formate: 2.73<br>Glycolate: 0.802<br>GAld dimer: 0.119	24	
	Pre-treated PET	Natural sunlight	14	1	2.29	Formate: 2.14<br>Glycolate: 0.882<br>GAld dimer: 0.125	21 (3 days)[c]	
TiO_2|Rh	EG	300 W Xe lamp	7	0.075	1.76	CO_2: 0.637<br>Formaldehyde: 0.531<br>Glycolaldehyde: 0.106<br>Acetaldehyde: 0.048	12	39
TiO_2|Au	Pre-treated PET	60 mW cm^{-2} UV lamp	14	0.5	0.00065	-	48	40
TiO_2|Pt	EG	0.028 mW cm^{-2} UV LED array	7	0.040	9.64	CO_2: 2.58<br>Formaldehyde: 2.66<br>Glycolaldehyde: 3.13<br>Formic acid: 0.313	4	41
TiO_2|Pt	Pre-treated PET	UV LED array	14	0.030	0.6	Glycolaldehyde	8	42
Single-atom-Pt-loaded TiO_2	Pre-treated PET	300 W Xe lamp	3-4	0.010	0.052	Glyoxal: 0.054<br>Glyoxylate: 0.063<br>Acetate: 0.096<br>Lactate: 0.104	120	43
Dynamically stabilised atomic Pt-loaded TiO_2	Pre-treated PET	Natural sunlight	14	1	3.14	-	216 (26 days)[d]	20
TiO_2|Pd	EG	5 mW cm^{-2} UV lamp	7	0.0065	44.5	-	4	44
TiO_2|Ag-Pd	EG	5 mW cm^{-2} UV lamp	7	0.002	16.0	CO_2: 1.08	30	45
TiO_2|Pt cryoaerogel composite	Pre-treated PET	Xe lamp; 122 mW cm^{-2}	14	0.00026	3.45	-	168	46

NiMo-loaded CN_x-carbon nanotube hybrid	Pre-treated PET	Simulated sunlight; 95 mW cm^{-2}	14	0.010	0.09	Glyoxal<br>Glycolate	16	[47]
$CN_x\|Ni_2P$	Pre-treated PET	Simulated sunlight; 100 mW cm^{-2}	14	0.0025	0.052	-	20	[48]
$CN_x\|Ni_2P$	Pre-treated PET	Simulated sunlight; 100 mW cm^{-2}	6	0.0012	0.102	-	96	[24]
$g\text{-}C_3N_4\|Pt$	Pre-treated PET	Simulated sunlight; 100 mW cm^{-2}	14	0.100	7.33	Formate<br>Acetate<br>Glyoxal<br>Glycolate	192	[49]
$BiVO_4\|MoO_x$	Pre-treated PET	300 W Xe lamp	14	0.050	1.96	Formate: 0.29<br>Acetate: 0.19<br>Glycolate	25	[50]
$CdS\|CdO_x$	Pre-treated PET	Simulated sunlight; 100 mW cm^{-2}	14	1.4×10^{-7}	4.13	Formate<br>Acetate<br>Glycolate<br>Lactate<br>Ethanol	144	[51]

[a] Reaction scale tabulated in m^2 for PC sheet systems and g_{cat} for suspended systems.

[b] Production rate tabulated on areal and g_{cat} basis for PC sheet and suspended systems, respectively. Values as reported converted to similar units where sufficient information was available. Where products were reported qualitatively, the products were tabulated without their production rates.

[c] Outdoor experiment under natural sunlight was performed for 3 days. However, considering dark hours, the irradiation time was 7 h per day, for a total of 21 h.

[d] Outdoor experiment under natural sunlight was performed for 26 days. However, considering dark hours, the irradiation time was 12 h per day, for a total of 216 h.

Table S18. Values used in the feasibility study of the 1 m^2-scale system.[a,b,c]

Component	Per unit	Cost (£)	Ref	Embodied energy (MJ)	ref	Carbon footprint (kg CO_2eq)	ref
**Capital[d]**							
Acrylic cell chamber	m^2	57.05	52	822		54.9	
Gasket	m^2	25.13	54	331		11	
Aluminium support frame	m^2	63.30	55	1280		116	
Steel crossbeam support	m	8.94	56	51.4		4.2	
Timber base	m^3	30.22	57	15016		899	
Aluminium rectangular tube for reactor base	m	13.80	58	33.7	53	2.9	53
Steel tube for reactor base	m	3.95	59	51.4		4.2	
0.25 m^2 glass panels	unit	22.20	60	222		15.7	
Toggle clamps	unit	2.95	61	-		-	
Tube adaptors	unit	1.76	62	-		-	
PVC tubing	m	0.48	63	0.009	64	0.00036	64
Miscellaneous (fittings, bearings, wheels, etc)	-	93.08	-	-	-	-	-
Installation labour	m^2	29.30	65	-	-	-	-
**Operation/consumables**							
Al:$SrTiO_3$|Pd_{CR}[e]	g	0.011	-	0.49	-	0.060	-
KOH	g	0.0011	66	0.033	67	0.003	67
Waste PET bottles[f]	g	−0.0012[g]	68	-	-	-	-
H_2O	L	0.0011	69	0.008	70	0.00032	71
N_2 (for reactor purging)	L	0.0050	72	0.003	67	0.00054	
Energy	kWh	0.18	73	1.57		0.105	67
**Products[h]**							
H_2	g	0.0070	74	0.2	75	0.012	76
Formate	g	0.0140	77	0.055		0.0034	67
Glycolate	g	0.030	78	0.058		0.0096	79
GAld dimer	g	0.89	80	0.13	67	0.0073	
Ethylene glycol	g	0.0035	81	0.061		0.0029	67
Terephthalate	g	0.0031	82	0.086		0.0044	

[a] For accuracy, the scope of the feasibility study is limited to the actual operation of the photoreactor, i.e., the STV creation rate, EROI and carbon footprint were calculated based on the amount of product generated and the associated capital and operational costs in the actual as-conducted experiment.

[b] Currency conversion is based on rates on 11 September 2024.

[c] Transportation of materials is not considered.

[d] Capital costs are amortised over the estimated lifetime of the photoreactor, i.e., 500 days.

[e] See Table S19 below for values used in calculation of cost, embodied energy and carbon footprint of Al:$SrTiO_3$|Pd_{CR}.

[f] Negative emissions from avoiding landfilling or incineration of waste PET are not considered.

[g] Negative cost resulting from gate fee for receiving waste PET bottles.

[h] Separation of products is not considered. See Note S8.

Table S19. Values used in calculating the cost, embodied energy and carbon footprint per unit of Al:$SrTiO_3$|Pd_{CR}.

Chemical	Per unit	Cost (£)	Ref	Embodied energy (MJ)	ref	Carbon footprint (kg CO_2eq)	ref
Al_2O_3	g	0.0065	83	0.16	84	0.00071	85
$SrTiO_3$	g	0.0019	86	0.077	67	0.0074	67
$SrCl_2$•$6H_2O$	g	0.00061	87	0.028		0.0036	
Na_2PdCl_4	g	0.0025	88	0.0047		0.0012	67,89
Polyvinylpyrrolidone	g	0.00077	90	0.13		0.010	91
$NaBH_4$	g	0.00077	92	0.045		0.0036	67

Table S20. Individual component contributions to Al:$SrTiO_3$|Pd_{CR} cost, embodied energy and carbon footprint.

Chemical	Quantity	Unit	Cost (£)	Embodied energy (MJ)	Carbon footprint (kg CO_2eq)
Al_2O_3	0.011	g	0.000072	0.0018	0.0000078
$SrTiO_3$	1	g	0.0019	0.077	0.0074
$SrCl_2$•$6H_2O$	14.5	g	0.0088	0.41	0.052
Na_2PdCl_4	0.028	g	0.000070	0.00013	0.000034
Polyvinylpyrrolidone	0.001	g	0.00000077	0.00013	0.0000074
$NaBH_4$	0.05	g	0.000039	0.0023	0.00018
Total	1	g	0.011	0.49	0.060

Table S21. Individual component contributions to capital, operation/consumables and product cost, embodied energy and carbon footprint.

Component	Quantity	Unit	Cost (£)	Embodied energy (MJ)	Carbon footprint (kg CO_2eq)
**Capital**					
Acrylic cell chamber	3.00	m^2	170.99	2463.70	164.55
Gasket	0.14	m^2	3.56	46.87	1.56
Aluminium support frame	0.21	m^2	13.44	271.87	24.64
Steel crossbeam support	2.36	m	21.10	121.30	9.91
Timber base	0.02	m^3	0.56	277.50	16.61
Aluminium rectangular tube for reactor base	4.20	m	57.96	141.54	12.18
Steel tube for reactor base	1.40	m	5.53	71.96	5.88
0.25 m^2 glass panels	4.00	unit	88.80	888	62.80
Toggle clamps	40	unit	118.00	0	0
Tube adaptors	10	unit	17.60	0	0
PVC tubing	4.72	m	2.27	0.042	0.0017
Miscellaneous (fittings, bearings, wheels, etc)	-	-	93.08	0	0
Installation labour	1.69	m^2	49.52	0	0
Total			642.40	4282.78	298.13
**Operation/consumables**					
Al:$SrTiO_3$|Pd	1.19	g	0.013	0.58	0.071
KOH	1122.00	g	1.23	37.03	3.37
Waste PET bottles	223.90	g	−0.27	0	0
H_2O	20.00	L	0.022	0.16	0.0064
N_2 (for reactor purging)	50.00	L	0.25	0.15	0.027
Energy[a]	8.47	kWh	1.53	13.30	0.89
Total			2.78	51.21141	4.36
**Products**					
H_2	0.097	g	0.00068	0.019	0.0012
Formate	2.07	g	0.029	0.11	0.0070
Glycolate	1.41	g	0.042	0.082	0.014
GAld dimer	0.32	g	0.28	0.042	0.0023
Ethylene glycol	38.40	g	0.13	2.34	0.11
Terephthalic acid	103.00	g	0.32	8.86	0.45
Total			0.81	11.46	0.59

[a] Energy consumed during PC synthesis. Measured using an energy meter.

Table S22. Parameters used in "pessimistic", "base" and "optimistic" cases in the sensitivity analysis of the 1 m^2-scale system. The base case parameters are the real conditions in which the 1 m^2 demonstration was performed.

Variable	Unit	Pessimistic case	Base case	Optimistic case
Catalyst reuse	days	1	3	10
Light intensity[a]	Sun	0.1	0.39[b]	2 (concentrated)
Daylight hours	hours	4	7	14
Reactor lifetime	days	300	500	1000
KOH concentration	M	10	4	0.5
EG source	-	Pristine PET	Waste PET bottles	Effluent waste[c]

[a] H_2 production was assumed to scale with the square root of light intensity.[93]

[b] Average measured light intensity over the duration of the 1 m^2 demonstration.

[c] Such as EG-rich discharge from various industries.[94–96]

Table S23. Results of the sensitivity analysis on the STV creation rate of the overall waste PET photoreforming process. Calculated based on the parameters described in Table S22.

STV creation rate (£ m^{-2} h^{-1})	Pessimistic case	Base case	Optimistic case
Catalyst reuse	−0.17	−0.15	−0.12
Light intensity	−0.16	−0.15	−0.13
Daylight hours	−0.16	−0.15	−0.14
Reactor lifetime	−0.20	−0.15	−0.12
KOH concentration	−0.24	−0.15	−0.10
EG source	−0.17	−0.15	−0.13

Table S24. Results of the sensitivity analysis on the EROI of the overall waste PET photoreforming process. Calculated based on the parameters described in Table S22.

EROI	Pessimistic case	Base case	Optimistic case
Catalyst reuse	0.19	0.19	0.20
Light intensity	0.19	0.19	0.20
Daylight hours	0.19	0.19	0.20
Reactor lifetime	0.18	0.19	0.21
KOH concentration	0.10	0.19	0.42
EG source	0.19	0.19	0.01

Table S25. Results of the sensitivity analysis on the carbon footprint of the overall waste PET photoreforming process. Calculated based on the parameters described in Table S22.

Carbon footprint (kg CO_2eq $MJ_{products}^{-1}$)	Pessimistic case	Base case	Optimistic case
Catalyst reuse	0.44	0.43	0.41
Light intensity	0.44	0.43	0.42
Daylight hours	0.44	0.43	0.42
Reactor lifetime	0.47	0.43	0.41
KOH concentration	0.87	0.43	0.18
EG source	0.43	0.43	6.18

Table S26. Results of the sensitivity analysis on STV creation rate considering only photocatalytic aspects (i.e., without considering waste PET pre-treatment). Calculated based on the parameters described in Table S22.

STV creation rate (£ m^{-2} h^{-1})	Pessimistic case	Base case	Optimistic case
Catalyst reuse	−0.14	−0.13	−0.09
Light intensity	−0.14	−0.13	−0.11
Daylight hours	−0.14	−0.13	−0.11
Reactor lifetime	−0.17	−0.13	−0.10

Table S27. Results of the sensitivity analysis on EROI considering only photocatalytic aspects (i.e., without considering waste PET pre-treatment). Calculated based on the parameters described in Table S22.

EROI	Pessimistic case	Base case	Optimistic case
Catalyst reuse	0.0038	0.011	0.038
Light intensity	0.0057	0.011	0.026
Daylight hours	0.0065	0.011	0.023
Reactor lifetime	0.0090	0.011	0.014

Table S28. Results of the sensitivity analysis on carbon footprint considering only photocatalytic aspects (i.e., without considering waste PET pre-treatment). Calculated based on the parameters described in Table S22.

Carbon footprint (kg CO_2eq $MJ_{products}^{-1}$)	Pessimistic case	Base case	Optimistic case
Catalyst reuse	18.5	6.18	1.85
Light intensity	12.2	6.18	2.73
Daylight hours	10.8	6.18	3.09
Reactor lifetime	7.73	6.18	5.02

Table S29. Results of the sensitivity analysis on STV creation rate considering only photocatalytic aspects (i.e., without considering waste PET pre-treatment) and excluding capital costs. Calculated based on the parameters described in Table S22.

STV creation rate (£ m^{-2} h^{-1})	Pessimistic case	Base case	Optimistic case
Catalyst reuse	−0.081	−0.069	−0.030
Light intensity	−0.078	−0.069	−0.048
Daylight hours	−0.076	−0.069	−0.052

Table S30. Results of the sensitivity analysis on the STV creation rate of a hypothetical overall water splitting system. Calculated based on the parameters described in Table S22.

STV creation rate (£ m^{-2} h^{-1})	Pessimistic case	Base case	Optimistic case
Catalyst reuse	−0.15	−0.15	−0.15
Light intensity	−0.15	−0.15	−0.15
Daylight hours	−0.15	−0.15	−0.15
Reactor lifetime	−0.19	−0.15	−0.12

Table S31. Results of the sensitivity analysis on the EROI of a hypothetical overall water splitting system. Calculated based on the parameters described in Table S22.

EROI	Pessimistic case	Base case	Optimistic case
Catalyst reuse	0.00097	0.0029	0.0097
Light intensity	0.00145	0.0029	0.0066
Daylight hours	0.0017	0.0029	0.0058
Reactor lifetime	0.0018	0.0029	0.0053

Table S32. Results of the sensitivity analysis on the carbon footprint of a hypothetical overall water splitting system. Calculated based on the parameters described in Table S22.

Carbon footprint (kg CO_2eq $MJ_{products}^{-1}$)	Pessimistic case	Base case	Optimistic case
Catalyst reuse	170.7	56.9	17.1
Light intensity	112.4	56.9	25.1
Daylight hours	99.6	56.9	28.5
Reactor lifetime	71.1	56.9	46.2

Table S33. Possible EG oxidation products and number of electrons required for their formation.[97]

Product	Number of electrons involved (per molecule)
GAld	2
Formaldehyde	1
Glycolic acid	4
Glyoxal	4
Glyoxylic acid	6
Formic acid	3
Oxalic acid	8
Carbon dioxide	5

Table S34. Charge consumed in the oxidation and reduction reaction in experiments on Al:$SrTiO_3$|Pd_{CR} and Al:$SrTiO_3$|Pd_{PR} PC sheets. Photocatalytic experiments were performed under AM1.5G illumination for 24 h at 25 °C with stirring.

Sample	Charged consumed (µmol cm^{-2})	
	Electrons	Holes
Al:$SrTiO_3$|Pd_{CR}	25.8±4.5	34.2±3.1
Al:$SrTiO_3$|Pd_{PR}	12.1±2.1	5.0±1.6

Table S35. Performance of Al:$SrTiO_3$|Pd_{CR} PC powder in a suspended system. The photocatalytic experiments were performed under AM1.5G illumination for 24 h at 25 °C with stirring.

Unit	H_2	Formate	Glycolate	GAld dimer
µmol	8.6±2.5	3.2±0.2	1.0±0.12	0.1±0.1
mmol g_{cat}^{-1} h^{-1}	0.2±0.1	0.1±0.003	0.02±0.004	0.003±0.002

Supplementary References

1 T. Kato, Y. Hakari, S. Ikeda, Q. Jia, A. Iwase and A. Kudo, *J. Phys. Chem. Lett.*, 2015, **6**, 1042–1047.

2 L. Jiang, J. Zhang, J. Chen, C. Dong, H. Tan, Q. Chen, J. Zhang, X. Chen and R. Wang, *Small*, DOI:10.1002/smll.202403636.

3 T. W. Kim and K.-S. Choi, *J. Phys. Chem. Lett.*, 2016, **7**, 447–451.

4 A. Srinivasan and M. Miyauchi, *J. Phys. Chem. C*, 2012, **116**, 15421–15426.

5 H. Lyu, T. Hisatomi, Y. Goto, M. Yoshida, T. Higashi, M. Katayama, T. Takata, T. Minegishi, H. Nishiyama, T. Yamada, Y. Sakata, K. Asakura and K. Domen, *Chem. Sci.*, 2019, **10**, 3196–3201.

6 H. Nishiyama, T. Yamada, M. Nakabayashi, Y. Maehara, M. Yamaguchi, Y. Kuromiya, Y. Nagatsuma, H. Tokudome, S. Akiyama, T. Watanabe, R. Narushima, S. Okunaka, N. Shibata, T. Takata, T. Hisatomi and K. Domen, *Nature*, 2021, **598**, 304–307.

7 Y. Ham, T. Hisatomi, Y. Goto, Y. Moriya, Y. Sakata, A. Yamakata, J. Kubota and K. Domen, *J. Mater. Chem. A*, 2016, **4**, 3027–3033.

8 Z. Zhao, E. J. Willard, H. Li, Z. Wu, R. H. R. Castro and F. E. Osterloh, *J. Mater. Chem. A*, 2018, **6**, 16170–16176.

9 T. Takata and K. Domen, *J. Phys. Chem. Lett. C*, 2009, **113**, 19386–19388.

10 T. Takata, J. Jiang, Y. Sakata, M. Nakabayashi, N. Shibata, V. Nandal, K. Seki, T. Hisatomi and K. Domen, *Nature*, DOI:10.1038/s41586-020-2278-9.

11 Y. Goto, T. Hisatomi, Q. Wang, T. Higashi, K. Ishikiriyama, T. Maeda, Y. Sakata, S. Okunaka, H. Tokudome, M. Katayama, S. Akiyama, H. Nishiyama, Y. Inoue, T. Takewaki, T. Setoyama, T. Minegishi, T. Takata, T. Yamada and K. Domen, *Joule*, 2018, **2**, 509–520.

12 T. Uekert, M. A. Bajada, T. Schubert, C. M. Pichler and E. Reisner, *ChemSusChem*, 2021, **14**, 4190–4197.

13 M. Schröder, K. Kailasam, J. Borgmeyer, M. Neumann, A. Thomas, R. Schomäcker and M. Schwarze, *Energy Technol.*, 2015, **3**, 1014–1017.

14 M. Rahaman, A. Dutta and P. Broekmann, *ChemSusChem*, 2017, **10**, 1733–1741.

15 H. Chen, Z. Guo, X. Liang, M.-Q. Zhang, M. Wang and D. Ma, *ACS Catal.*, 2025, **15**, 6287–6295.

16 A. Dailey, J. Shin and C. Korzeniewski, *Electrochim. Acta*, 1998, **44**, 1147–1152.

17 R. G. Da Silva, A. Rodrigues de Andrade, K. Servat, C. Morais, T. W. Napporn and K. B. Kokoh, *ChemElectroChem*, 2020, **7**, 4326–4335.

18 H.-Z. Ma, S.-H. He, Y. Zhang, L. Wang, Y.-N. Yi and Y.-Y. Yang, *ACS Sustain. Chem. Eng.*, 2024, **12**, 12249–12259.

19 A. Bin Mohamad Annuar, Y. Liu, S. Bhattacharjee, J. Slaughter, I. Mikulska, F. N. Sayed, C. P. Grey, D. S. Wright and E. Reisner, *Nat. Chem. Eng.*, DOI:10.1038/s44286-026-00406-y.

20 W. H. Lee, H. Park, C. W. Lee, H. Kim, J. H. Jeong, J. I. Yun, S.-U. Bang, J. Heo, K. H. Ahn, G. D. Cha, M. S. Bootharaju, B.-H. Lee, J. Ryu, M. Kim, T. Hyeon and D.-H. Kim, *Nat. Nanotechnol.*, 2025, **20**, 1237–1246.

21 K. Roh, A. Bardow, D. Bongartz, J. Burre, W. Chung, S. Deutz, D. Han, M. Heßelmann, Y. Kohlhaas, A. König, J. S. Lee, R. Meys, S. Völker, M. Wessling, J. H. Lee and A. Mitsos, *Green Chem.*, 2020, **22**, 3842–3859.

22 T. Uekert, C. M. Pichler, T. Schubert and E. Reisner, *Nat. Sustain.*, 2020, **4**, 383–391.

23 W. Zhang, X. Hao, X. Liu, M. Chu, S. Li, X. Wang, F. Jiang, L. Wang, Q. Zhang, J. Chen, D. Wang and M. Cao, *Angew. Chemie Int. Ed.*, DOI:10.1002/anie.202500814.

24 S. Bhattacharjee, C. Guo, E. Lam, J. M. Holstein, M. Rangel Pereira, C. M. Pichler, C. Pornrungroj, M. Rahaman, T. Uekert, F. Hollfelder and E. Reisner, *J. Am. Chem. Soc.*, 2023, **145**, 20355–20364.

25 B. A. Pinaud, J. D. Benck, L. C. Seitz, A. J. Forman, Z. Chen, T. G. Deutsch, B. D. James, K. N. Baum, G. N. Baum, S. Ardo, H. Wang and T. F. Jaramillo, *Energy Environ. Sci.*, 2013, **6**, 1983–2002.

26 C. Y. Toe, J. Pan, J. Scott and R. Amal, *ACS ES&T Eng.*, 2022, **2**, 1130–1143.

27 L. S. F. Frowijn and W. G. J. H. M. van Sark, *Sustain. Energy Technol. Assessments*, 2021, **48**, 101631.

28 A. Barredo, A. Asueta, I. Amundarain, J. Leivar, R. Miguel-Fernández, S. Arnaiz, E. Epelde, R. López-Fonseca and J. I. Gutiérrez-Ortiz, *J. Environ. Chem. Eng.*, 2023, **11**, 109823.

29 L. Yan, P. M. Witt, T. F. Edgar and M. Baldea, *Ind. Eng. Chem. Res.*, 2021, **60**, 3027–3037.

30 A. T. Laitinen, V. M. Parsana, O. Jauhiainen, M. Huotari, L. J. P. van den Broeke, W. de Jong, T. J. H. Vlugt and M. Ramdin, *Ind. Eng. Chem. Res.*, 2021, **60**, 5588–5599.

31 D. Núñez, P. Oulego, S. Collado, F. A. Riera and M. Díaz, *Sep. Purif. Technol.*, 2022, **284**, 120274.

32 K. Chen, S. Hao, H. Lyu, G. Luo, S. Zhang and J. Chen, *Sep. Purif. Technol.*, 2017, **172**, 100–106.

33 H. Wu, L. Valentino, S. Riggio, M. Holtzapple and M. Urgun-Demirtas, *Sep. Purif. Technol.*, 2021, **265**, 118108.

34 L. Wang, H. Meng, P. K. Shen, C. Bianchini, F. Vizza and Z. Wei, *Phys. Chem. Chem. Phys.*, 2011, **13**, 2667–2673.

35 X. Zhu, T. Zhang, D. Jiang, H. Duan, Z. Sun, M. Zhang, H. Jin, R. Guan, Y. Liu, M. Chen, H. Ji, P. Du, W. Yan, S. Wei, Y. Lu and S. Yang, *Nat. Commun.*, 2018, **9**, 4177.

36 Z. Yu and S. Chuang, *J. Catal.*, 2007, **246**, 118–126.

37 F. Li, Q. Gu, Y. Niu, R. Wang, Y. Tong, S. Zhu, H. Zhang, Z. Zhang and X. Wang, *Appl. Surf. Sci.*, 2017, **391**, 251–258.

38 X.-Y. Ma, H.-Z. Ma, S.-H. He, Y. Zhang, Y.-N. Yi and Y.-Y. Yang, *Mater. Today Phys.*, 2023, **37**, 101191.

39 T. F. Berto, K. E. Sanwald, W. Eisenreich, O. Y. Gutiérrez and J. A. Lercher, *J. Catal.*, 2016, **338**, 68–81.

40 E. M. N. Thiloka Edirisooriya, P. S. Senanayake, P. Xu and H. Wang, *J. Environ. Chem. Eng.*, 2023, **11**, 111429.

41 L. Roebuck, H. Daly, L. Lan, J. Parker, A. Gostick, N. Skillen, S. J. Haigh, M. Falkowska and C. Hardacre, *J. Catal.*, 2025, **442**, 115876.

42 L. Roebuck, M. Hu, H. Daly, H. Warsahartana, L. S. Natrajan, A. Garforth, C. D'Agostino, M. Falkowska and C. Hardacre, *Catal. Today*, 2025, **452**, 115242.

43 X. Han, M. Jiang, H. Li, R. Li, N. H. M. Sulaiman, T. Zhang, H. Li, L. Zheng, J. Wei, L. He and X. Zhou, *J. Colloid Interface Sci.*, 2024, **665**, 204–218.

44 Z. H. N. Al-Azri, W.-T. Chen, A. Chan, V. Jovic, T. Ina, H. Idriss and G. I. N. Waterhouse, *J. Catal.*, 2015, **329**, 355–367.

45 A. K. Wahab, M. A. Nadeem and H. Idriss, *Front. Chem.*, DOI:10.3389/fchem.2019.00780.

46 W. H. Lee, C. W. Lee, G. D. Cha, B.-H. Lee, J. H. Jeong, H. Park, J. Heo, M. S. Bootharaju, S.-H. Sunwoo, J. H. Kim, K. H. Ahn, D.-H. Kim and T. Hyeon, *Nat. Nanotechnol.*, DOI:10.1038/s41565-023-01385-4.

47 X. Gong, F. Tong, F. Ma, Y. Zhang, P. Zhou, Z. Wang, Y. Liu, P. Wang, H. Cheng, Y. Dai, Z. Zheng and B. Huang, *Appl. Catal. B Environ.*, 2022, **307**, 121143.

48 T. Uekert, M. A. Bajada, T. Schubert, C. M. Pichler and E. Reisner, *ChemSusChem*, 2021, **14**, 4190–4197.

49 T. K. A. Nguyen, T. Trần-Phú, X. M. C. Ta, T. N. Truong, J. Leverett, R. Daiyan, R. Amal and A. Tricoli, *Small Methods*, DOI:10.1002/smtd.202300427.

50 X. Liang, T. Gao, Y. Cui, Q. Dong, X. Li, A. Labidi, E. Lichtfouse, F. Li, F. Yu and C. Wang, *Appl. Catal. B Environ. Energy*, 2024, **357**, 124326.

51 T. Uekert, M. F. Kuehnel and W. Wakerley, *Energy Environ. Sci.*, 2018, **11**, 2853–2857.

52 Plastic Sheets Direct, 5 mm Clear Acrylic, https://www.plasticsheetsdirect.co.uk/product/5mm-clear-acrylic/, (accessed 9 September 2024).

53 Australian Research Council, *Environmental Performance in Construction*, 2019.

54 Rubber Co, Heavy duty rubber sheet, https://rubberco.co.uk/products/commercial-rubber-sheet-linear-meter, (accessed 11 September 2024).

55 Aluminium Online, Aluminium Plain Sheet, https://www.aluminium-online.co.uk/product-category/sheet/aluminium-sheet/, (accessed 9 September 2024).

56 KI Metals, Mild steel U channel, https://kimetals.co.uk/materials/mild-steel/mild-steel-channel/kim41734/, (accessed 11 September 2024).

57 The Research Agency of the Forrestry Commission, *Timber Price Indices*, Midlothian, 2024.

58 Aluminium Warehouse, Aluminium rectangular tube,

https://www.aluminiumwarehouse.co.uk/products/40-mm-x-20-mm-x-2-mm-aluminium-rectangular-tube, (accessed 11 September 2024).

59 Metals4u, Mild steel tube, https://www.metals4u.co.uk/materials/mild-steel/mild-steel-tube/tube/2337-p, (accessed 11 September 2024).

60 The Glass Warehouse, Frosted Satin Glass, https://www.theglasswarehouse.co.uk/frosted-satin-glass/, (accessed 9 September 2024).

61 Adafruit Industries LLC, Toggle clamp - rubber tip, https://www.digikey.co.uk/en/products/detail/adafruit-industries-llc/2456/7244953, (accessed 11 September 2024).

62 RS Pro, Straight tube-to-tube adaptor, https://uk.rs-online.com/web/p/pneumatic-fittings/9160886, (accessed 11 September 2024).

63 Auto Silicone Hoses, PVC Unreinforced Tube, https://www.autosiliconehoses.com/pvc-tube-6mm-1-4-inch-metre-clear.html, (accessed 9 September 2024).

64 M. F. Ashby, *Materials and the Environment: Eco-informed Material Choice*, 2009.

65 M. R. Shaner, H. A. Atwater, N. S. Lewis and E. W. McFarland, *Energy Environ. Sci.*, 2016, **9**, 2354–2371.

66 Finox Pellets Industries, Potassium hydroxide pellets, LR, https://www.finoxpellets.in/caustic-potash.html, (accessed 14 April 2025).

67 B. Wernet, G., Bauer, C., Steubing, B., Reinhard, J., Moreno-Ruiz, E., and Weidema, *Int. J. Life Cycle Assess.*, 2016, **21**, 1218–1230.

68 European Environmental Agency, *Investigating Europe's secondary raw material markets*, Copenhagen, 2022.

69 Cambridge Water, Metered water charges 2024-2025, https://www.cambridge-water.co.uk/household/my-bills-and-payments/my-bill-explained/metered-charges-explained/metered-charges, (accessed 11 September 2024).

70 W. Mo, Q. Zhang, J. R. Mihelcic and D. R. Hokanson, *Water Res.*, 2011, **45**, 5577–5586.

71 No Title, https://www.winnipeg.ca/finance/findata/matmgt/documents/2012/682-2012/682-%0A2012_Appendix_H-WSTP_South_End_Plant_Process_Selection_Report/Appendix 7.pdf.

72 BOC Online UK, Nitrogen (Oxygen Free) 230bar Cylinder, https://www.boconline.co.uk/shop/en/uk/nitrogen-oxygen-free-230-bar-cylinder, (accessed 9 September 2024).

73 Department for Energy Security & Net Zero, *Quarterly Energy Prices: UK October to December 2024 and annual date for 2024*, 2025.

74 R. Winterbourne, Hydrogen Production Costs: A Comparison of Green, Blue, and Grey Hydrogen, https://haush.co.uk/hydrogen-production-costs-a-comparison-of-green-blue-and-grey-hydrogen/#:~:text=In the UK%2C grey hydrogen,for the UK’s decarbonisation strategy.

75 O. Massarweh, M. Al-khuzaei, M. Al-Shafi, Y. Bicer and A. S. Abushaikha, *J. CO2 Util.*, 2023, **70**, 102438.

76 E. Lewis, S. McNaul, M. Jamieson, M. Henriksen, H. Matthews, L. Walsh, J. Grove, T. Shultz, T. Skone and R. Stevens, *Comparison of Commercial, State-of-the-Art, Fossil-Based Hydrogen Production Technologies*, 2022.

77 Penta Chemicals Unlimited, Formic acid 98%, https://www.pentachemicals.eu/en/chemicals/formic-acid-98-298, (accessed 22 April 2025).

78 Shaanxi Lifecare Biotechnology Co. Ltd., Glycolic acid 99%, https://www.alibaba.com/product-detail/Glycolic-Acid-Powder-Cosmetic-Raw-Materials_1600796035562.html, (accessed 22 April 2025).

79 I. Dincer, M. A. Rosen and M. Al-Zareer, in *Comprehensive Energy Systems*, Elsevier, 2018, pp. 470–520.

80 SelleckChem, Glycolaldehyde dimer, https://www.selleckchem.com/products/glycolaldehyde-dimer.html, (accessed 22 April 2025).

81 ReAgent, Ethylene glycol, https://www.chemicals.co.uk/ethylene-glycol, (accessed 22 April 2025).

82 Shanghai Topfine Chemical Co. Ltd., Terephthalic acid powder, https://www.alibaba.com/product-detail/Industrial-Grade-Terephthalic-Acid-Powder-Organic_1601422498107.html, (accessed 22 April 2025).

83 Luoyang Tongrun Info Technology Co. Ltd., Al_2O_3 powder, 50nm, https://www.alibaba.com/product-detail/99-999-High-Purity-Gamma-Alumina_1601242621394.html, (accessed 11 September 2024).

84 United Nations Environment Programme, *Environmental Risks and Challenges of Anthropogenic Metals Flows and Cycles*, 2013.

85 L. Edwards, M. Hunt, P. Weyell, J. Nord, J. Côté, P. Coulombe and N. Morais, *JOM*, 2022, **74**, 4909–4919.

86 Jinan Yudong Trading Co. Ltd., $SrTiO_3$ powder nanoparticles, https://www.alibaba.com/product-detail/Strontium-Titanate-Powder-Nanoparticles-Srtio3-Nanopowder_1601169857614.html, (accessed 11 September 2024).

87 Jinan Jinbang Chemical Co. Ltd., $SrCl_2.6H_2O$, 99%.

88 Jinan Future Chemical Co. Ltd., Sodium tetrachloropalladate (II), https://www.alibaba.com/product-detail/High-Quality-Na2PdCl4-for-Catalyst-Sodium_1600258124775.html, (accessed 14 April 2025).

89 CarbonCloud, *Technical Report - Climate Footprint of Salt (NaCl)*, 2025.

90 Shanghai Kean Technology Co. Ltd., Polyvinylpyrrolidone K30, https://www.alibaba.com/product-detail/Factory-Supply-Polyvidone-PVP-K30-Polyvinylpyrrolidone_1600689428848.html, (accessed 14 April 2025).

91 Ecovamed, *Carbon footprint evaluation of PVP (povidone), a key excipient for medicines*, 2023.

92 Henan Zhengzhou Yichi Technology Co. Ltd., Sodium borohydride, https://www.globalsources.com/Fine-chemical/16940-66-2-1224471430p.htm, (accessed 14 April 2025).

93 Y. Nosaka and A. Y. Nosaka, *J. Phys. Chem. C*, 2018, **122**, 28748–28756.

94 E. C. Wood, W. B. Knighton, E. C. Fortner, S. C. Herndon, T. B. Onasch, J. P. Franklin, D. R. Worsnop, T. R. Dallmann, D. R. Gentner, A. H. Goldstein and R. A. Harley, *Environ. Sci. Technol.*, 2015, **49**, 3322–3329.

95 C. A. Staples, J. B. Williams, G. R. Craig and K. M. Roberts, *Chemosphere*, 2001, **43**, 377–383.

96 K. Verschueren, *Handbook of environmental data on organic chemicals: Vol.1*, John Wiley and Sons, Inc, New York, 2001.

97 T. Matsumoto, M. Sadakiyo, M. L. Ooi, S. Kitano, T. Yamamoto, S. Matsumura, K. Kato, T. Takeguchi and M. Yamauchi, *Sci. Rep.*, 2014, **4**, 5620.

End of Supplementary Information
